# Dyconnmap: Dynamic connectome mapping—A neuroimaging python module

**DOI:** 10.1002/hbm.25589

**Published:** 2021-07-11

**Authors:** Avraam D. Marimpis, Stavros I. Dimitriadis, Rainer Goebel

**Affiliations:** ^1^ Cognitive Neuroscience Department, Faculty of Psychology and Neuroscience Maastricht University Maastricht The Netherlands; ^2^ Neuroinformatics Group, Cardiff University Brain Research Imaging Center (CUBRIC) School of Psychology, Cardiff University Cardiff United Kingdom; ^3^ Brain Innovation B.V Maastricht The Netherlands; ^4^ Institute of Psychological Medicine and Clinical Neurosciences Cardiff University School of Medicine Cardiff United Kingdom; ^5^ Cardiff University Brain Research Imaging Center (CUBRIC) School of Psychology, Cardiff University Cardiff United Kingdom; ^6^ School of Psychology Cardiff University Cardiff United Kingdom; ^7^ Neuroscience and Mental Health Research Institute Cardiff University Cardiff United Kingdom; ^8^ MRC Centre for Neuropsychiatric Genetics and Genomics School of Medicine, Cardiff University Cardiff United Kingdom

**Keywords:** chronnectomics, complex networks, dynamic connectivity, EEG, fMRI, functional connectivity, graph theory, human connectome, MEG, python, statistical analysis

## Abstract

Despite recent progress in the analysis of neuroimaging data sets, our comprehension of the main mechanisms and principles which govern human brain cognition and function remains incomplete. Network neuroscience makes substantial efforts to manipulate these challenges and provide real answers. For the last decade, researchers have been modelling brain structure and function via a graph or network that comprises brain regions that are either anatomically connected via tracts or functionally via a more extensive repertoire of functional associations. Network neuroscience is a relatively new multidisciplinary scientific avenue of the study of complex systems by pursuing novel ways to analyze, map, store and model the essential elements and their interactions in complex neurobiological systems, particularly the human brain, the most complex system in nature. Due to a rapid expansion of neuroimaging data sets' size and complexity, it is essential to propose and adopt new empirical tools to track dynamic patterns between neurons and brain areas and create comprehensive maps. In recent years, there is a rapid growth of scientific interest in moving functional neuroimaging analysis beyond simplified group or time‐averaged approaches and sophisticated algorithms that can capture the time‐varying properties of functional connectivity. We describe algorithms and network metrics that can capture the dynamic evolution of functional connectivity under this perspective. We adopt the word ‘chronnectome’ (integration of the Greek word ‘Chronos’, which means time, and connectome) to describe this specific branch of network neuroscience that explores how mutually informed brain activity correlates across time and brain space in a functional way. We also describe how good temporal mining of temporally evolved dynamic functional networks could give rise to the detection of specific brain states over which our brain evolved. This characteristic supports our complex human mind. The temporal evolution of these brain states and well‐known network metrics could give rise to new analytic trends. Functional brain networks could also increase the multi‐faced nature of the dynamic networks revealing complementary information. Finally, we describe a python module (https://github.com/makism/dyconnmap) which accompanies this article and contains a collection of dynamic complex network analytics and measures and demonstrates its great promise for the study of a healthy subject's repeated fMRI scans.

## Graphical abstract

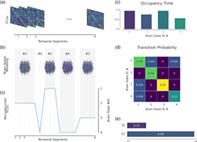

## 1 INTRODUCTION

An exciting and prominent approach in studying the brain's underlying processes and physiological activity is via neural synchronisation as observed among various brain regions. This representation is found to unveil both short and long‐range distance coordinated communication between neural populations during cognitive and perceptual tasks. Short‐range synchronisation refers to the observed synchrony between regions of the brain adjacent or related to a single stimulus, while long‐range to widely separated areas (see Penny, Friston, Ashburner, Kiebel, & Nichols, 2011 for an in‐depth analysis).

This synchronisation evokes complex brain networks both in structural and functional systems. Structural (also referred to as anatomical) connectivity implies the interconnection of cortical areas: tracts physically connect different brain areas. On the other hand, functional and effective connectivity is a byproduct of neuroimaging. There are multiple methods (and modalities) to approach functional connectivity. Common strategies include measuring the electrical activity observed in the cerebral cortex by using electroencephalography (EEG), detection of the changes in the blood flow with the usage of functional magnetic resonance imaging (fMRI) or recording the magnetic fields—produced by electrical currents which occur in the brain—by using magnetoencephalography (MEG). In any case, functional connectivity describes the statistical dependence between brain regions. In comparison, effective connectivity focuses on the causation of the connectivity between (or among) regions of interest and how they affect each other (e.g., the activation in one area may inhibit another one). For a more in‐depth discussion, it is highly recommended that the interested reader go through the published work of Bullmore and Sporns (2009) and Rubinov and Sporns (2010).

The most common approach to studying these networks is the static method. A network is constructed from the estimated connectivity, commonly using Pearson's correlation coefficient between the brain areas. However, this approach makes strong assumptions about the spatial and temporal stationarity throughout the measured period. In addition, a more substantial method is a dynamic approach. This approach became evident from a resting‐state (RS) fMRI study (Chang & Glover, 2010) in which the authors observed fluctuations over the functional connectivity over time; though it has been shown that this variability is not RS‐exclusive (Gonzalez‐Castillo et al., 2012; Kucyi, Salomons, & Davis, 2013). These findings steered a significant number of subsequent neuroimaging studies. This fact is partially due to the accessibility of this approach; the most well‐known strategy involves a *window sliding* over the time series and estimate of the correlation among them. This agglomeration of connectivity matrices make up the *dynamic functional connectome* (the dynamic functional connectome) (Preti, Bolton, & Van De Ville, 2017). Other approaches include more sophisticated methods and pipelines such as: using PCA to identify FC‐patterns (eigenconnectivities) (Leonardi et al., 2013), ICA‐derived time series (Allen et al., 2014), mutually temporally independent dynamic connectivity patterns (Yaesoubi, Miller, & Calhoun, 2015), data‐driven frameworks to capture time‐frequency information (Yaesoubi, Allen, Miller, & Calhoun, 2015) and the meta‐state framework (Miller et al., 2016) for defining and analyzing district states.

The developments around dynamic connectivity gave rise to a whole new field of study in neuroimaging. The so‐called ‘chronnectomics’ (Calhoun, Miller, Pearlson, & Adalı, 2014) (the junction of ‘Chronos’—the greek word of time, and ‘omics’ describes the efficacy of objects being categorised based on common traits) are purposed in capturing the time‐varying properties of connectivity in a spatio‐temporal manner. While the term connectome focuses on the brain's wiring (Sporns, Tononi, & Kötter, 2005), chronnectome is used to describe a focus on identifying time‐varying, but reoccurring, patterns of coupling among brain regions. A further notion that sparked from these engagements is the ‘dynamo’ (beyond the connectome). It is a framework that encapsulates and advances the connectome's notion to the underlying mechanisms involved in producing and processing signals within the brain.

The adoption of these newly founded frameworks was immediate across different topics and modalities. One of the first studies (Allen et al., 2014) introduced a complete methodological framework on the whole‐brain characterisation of regional differences in FC variability and discrete FC states. Exploiting the notion of dynamic connectivity & FC states, the authors in (Damaraju et al., 2014) found distinct differences within the networks among brain regions (hyperconnectivity and reduced connectivity) in patients suffering from schizophrenia and healthy participants. On the same topic, Cetin et al. (2016) combined fMRI/MEG to compare the static and dynamic connectivity and their discrimination efficacy between patients and healthy controls. Considering the FC states in the EEG field, Dimitriadis, Laskaris, Bitzidou, Tarnanas, and Tsolaki (2015) examined the developmental changes in the network organisation as observed during mental calculations and in resting‐state between children and young adults. The authors (Yu et al., 2016) did discover a different organisation in the networks between patients with the behavioural variant of frontotemporal dementia and patients with Alzheimer's, suggesting different pathophysiological mechanisms in these two separate neurodegenerative disorders. Zeng et al. (2015) proposed a biomarker that could be used to track the cognitive function of diabetic patients. They found that the FC is decreased in amnestic Mild Cognitive Impairment (aMCI) patients in specific frequency bands. These findings were also reflected in the organisation of the brain networks by using the relevant graph analysis.

A step further, and of equal importance are the studies focused on the reproducibility and replicability of conclusions drawn from other published studies. Allen and her colleagues (Allen, Damaraju, Eichele, Wu, & Calhoun, 2018) performed a cross‐modal EEG‐fMRI resting‐state study to replicate drowsiness effects in two conditions, eyes open and eyes closed, in a limited sample from their previous study. In (Abrol et al., 2017), the authors analyzed 7,500 human brain resting‐state fMRI scans and successfully replicated basic time‐varying FC states and state summary measures across subjects. For a comprehensive review, the interested reader is highly encouraged to consult the works of Preti et al. (2017) and Calhoun et al. (2014). In the last decade, Python has emerged as a highly suitable environment for analysing neuroimaging data and a direct replacement for MATLAB® (The Mathworks, Inc.) environment. Python is a high‐level, general‐purpose programming language that has dramatically attracted attention from the neuroimaging community. In this article, we present ‘dyconnmap’ (abbreviated from ‘dynamic connectome mapping’), a neuroimaging python module. This module is specifically designed to estimate the dynamic connectivity and analyse complex brain networks from neurophysiological data of any modality. It is organised in several submodules, chronnectomics and graph‐theoretical algorithms, (symbolic) time series and statistical methods. The module's development is an ongoing effort to extend it by adding more algorithms related to graph analysis, statistical approaches and increase transparency and ensure replicability. Considering the increasing acceptance and usage of Python in analysing neuroimaging data, we firmly believe that this module will be a great addition to every practitioner's toolbox engaged in brain connectivity analysis.

Multimodal neuroimaging has become a driving force of current neuroimaging research due to its clinical recognition in various brain diseases like Alzheimer's disease (Liu et al., 2014), schizophrenia (Cooper, Barker, Radua, Fusar‐Poli, & Lawrie, 2014) and so forth. Multimodal neuroimaging advances the fundamental neuroscience research by overcoming the knowledge extracted by a single modality and by untangling the associations of findings from more than one neuroimaging resource (Liu et al., 2015). Neuroscientists adopted modern network science tools to analyze brain networks constructed from most neuroimaging modalities, and they also developed novel network tools tailored to brain network neuroscience (Stam, 2014). Motivated by new findings of network neuroscience of high clinical importance with various functional neuroimaging modalities, we developed a module where someone can construct functional brain networks and can combine them, revealing their association and complementarity of the tabulated information. Our module is tailored to functional neuroimaging (EEG/MEG/ECoG/fMRI). At the same time, we provide a set of tools to compare structural brain networks with functional brain networks from various modality sources.

The layout of the article is as follows. In Sections 2 and 3, we will introduce functional brain networks and in both static and dynamic configurations. Subsequently, in Section 4, we will discuss how to use graphs to construct brain states. In Section 5, we will describe the different variants of bran networks. In Section 6, we will see how one can compare different networks using graph distances. In Section 7, we will dive into graph signal processing (GSP), and in Section 8, we will present the relationship between connectomics and chronnectomics. In the subsequent Sections 9 to 11, we will discuss a variety of applications. Finally, in Section 12, we will discuss our module and the motivation. We will close in Section 13 with a general discussion, thoughts and plans.

## 2 CONSTRUCTION OF FUNCTIONAL BRAIN NETWORKS

A network or graph is a well‐studied mathematical representation of many complex systems. It is defined by a set of nodes (vertices) and a collection of links (edges) between pairs of nodes. In a large‐scale brain network, nodes represent brain regions or anatomical parcellations (parcels), while links represent different types of connections like anatomical, functional or effective connections (Friston, 2011). A graph representing streamline‐based connectivity among regions of interest can be created by anatomical connections using diffusion‐weighted data and tractography. The general principle is to seed within regions of interest (ROIs), or across the whole brain, identified sets of streamlines that connect pairs of anatomical regions according to an atlas. Then, one can use the properties of these streamlines to derive a quantitative measure of region‐to‐region connectivity (Chung et al., 2017) or combine more than two into a single anatomical brain network that further improves the reliability of the derived network metrics (Dimitriadis et al., 2017; Messaritaki, Dimitriadis, & Jones, 2019).

The functional brain networks represent patterns of cross‐correlations between electro‐magneto‐encephalographic (EEG; MEG) and BOLD signals (fMRI), quantified from these temporal fluctuations by adopting proper connectivity estimators, like correlation, partial correlation, mutual information, phase locking values (PLV) (Lachaux, Rodriguez, Martinerie, & Varela, 1999), phase lag index (PLI) (Stam, Nolte, & Daffertshofer, 2007), the imaginary part of phase locking values (iPLV) (Dimitriadis et al., 2015). The effective brain network represents functional patterns of causal interactions—as computed with conditional mutual information, transfer entropy—which correspond to vital connectivity measures of directed information flow. All these types of networks are represented by their connectivity (adjacency) 2D matrices. Rows and columns in these matrices denote brain areas (nodes), while matrix entries denote links between pairs of brain areas. Any type of estimation over these brain networks remains unaltered by the ordering of nodes, although the visualisation is affected.

### 2.1 Definition of nodes in brain networks

The nature of brain nodes and the relevant links in subject‐specific brain networks is determined by the adaptation of brain mapping methods of an atlas that drives the parcellation scheme and estimators of functional connectivity. Every possible combination of the choices mentioned above could lead to an extensive repertoire of experimental setups (Horwitz, 2003; Dimitriadis et al., 2018).

We cannot stress enough that neuroscientists carefully choose the right combination because the nature of both nodes and functional links shape the neurobiological interpretation of a subject's cognition, health status and the overall network topology (Butts, 2009). Parcellation schemes ideally smooth brain areas heterogeneously into single ones in a one‐to‐one mapping. More often, they divide the brain cortex into non‐spatially overlapped brain areas. Similarly, the source localisation of brain activity recorded with MEG and EEG sensors leads to similar brain mapping using famous source localisation algorithms (Van Veen, van Dronglen, Yuchtman, & Suzuki, 1997). However, in general, the EEG and MEG sources could not be spatially aligned with brain areas' boundaries (Ioannides, 2007).

### 2.2 Definition of functional links in brain networks

Additionally, on the definition of brain nodes, functional links could be classified regarding their functional coupling strength and the existence or lack therefore of directionality, based on the adaptation of the relevant connectivity methodology. Brain networks with binary links capture the presence or absence of connections, while weighted brain networks also keep the existing links' functional coupling strength. In anatomical networks, weights may represent any feature related to anatomical tracts like fractional anisotropy (FA) and mean diffusivity (MD) (Dimitriadis, Sallis, Tarnanas, & Linden, 2017). Simultaneously, weights in functional and effective brain networks may represent the magnitude of functional association or causal interactions, respectively. Many studies eliminate the functional weights by applying arbitrary thresholding schemes, like absolute or proportional thresholds. This is a substantial drawback in neuroimaging analysis under a network neuroscience framework that leads to non‐reproducible results (van den Heuvel et al., 2017). Recently, a data‐driven topological filtering method has been introduced called orthogonal minimal spanning tree (OMST) (Dimitriadis, Antonakakis, Simos, Fletcher, & Papanicolaou, 2017; Dimitriadis, Drakesmith, et al., 2017), which provides denser brain networks than the minimal spanning tree (MST) (Tewarie, van Dellen, Hillebrand, & Stam, 2015).

Functional links may also be differentiated by the presence or absence of directionality adopting proper connectivity estimators, like transfer entropy, conditional mutual information, Granger causality. Effective functional connections may conceptually be represented with directed links. In contrast, effective anatomical connections are not a valid representation proved by tract‐tracing studies that indicate the existence of a substantial portion of reciprocal structural connections between many brain areas in the cortex.

### 2.3 Evaluation of functional/effective connectivity with various measures

Functional connections correspond to magnitudes of temporal correlations in activity and may occur between pairs of anatomically unconnected regions (Friston, 2011). Depending on the measure, functional connectivity may reflect linear or non‐linear interactions, based on the amplitude (correlation, partial correlation), on the spectrum (coherence) or in phase domain (PLV, PLI, wPLI) (Zhou, Thompson, & Siegle, 2009). Effective connections represent direct or indirect causal influences of one brain area into another and may be estimated from observed from either directly from empirical data with transfer entropy, conditional mutual information, Granger causality (Lindner, Vicente, Priesemann, & Wibral, 2011) or model‐based like DCM or SPM (Friston, Harrison, & Penny, 2003). Apart from the within‐frequency functional connectivity estimators, the between‐frequency or cross‐frequency coupling (CFC) estimators are also important to quantify interactions among brain areas oscillating in different frequency. For further reading, see the next section, while for identifying ‘true’ functional/effective interactions in both within‐frequency and CFC, a surrogate analysis is important (see Section 3).

Many approaches have been proposed to reduce the leakage effect introduced after source localisation of brain activity. This approach involves the orthogonalisation of virtual time series in a bivariate or multivariate mode (Hipp, Hawellek, Corbetta, Siegel, & Engel, 2012; Brookes, Woolrich, & Barnes, 2012). A solution to ghost interactions has been introduced via a hyperedge bundling procedure (Palva et al., 2018; Wang et al., 2018), while a motif‐based have been proposed to reveal true CFC interactions (Siebenhühner et al., 2020). These methods will be future additions to our module.

### 2.4 Different types of CFC estimators

Electro‐ and MEG (EEG, MEG) is a complex signal enriched with different frequency components that interact with each other. Within‐frequency interactions include time‐frequency transformations via Hilbert, wavelet or Gabor transform. They can integrate amplitude, frequency or phase modulations between brain areas oscillating within‐the‐same‐frequency across experimental time. It is well‐known at the microscopic level, neuronal cell assemblies oscillate at different frequencies. Simultaneously, they synchronise their activity when different brain areas demand exchange, retrieval of information and a quick response to natural stimuli (Buzsáki & Draguhn, 2004). Thus, CFC is a substrate mechanism that bridges brain activity of distinct brain areas with a different frequency content supporting local and global processes and directly linked to the integration of distributed information.

Jensen and Colgin (2007) described different types of CFC interactions: (a) amplitude (power) to amplitude (power) (AAC), (b) phase to phase (PPC) and (c) phase to amplitude (power) (PAC). There is high evidence that phase‐to‐amplitude coupling occurs in both humans and animals in many brain structures, like in prefrontal cortices, in entorhinal, in the hippocampus and in a more distributed set of brain areas (Mormann et al., 2005; Cohen, 2008; Tort et al., 2008; Cohen et al., 2009,b; Axmacher et al. 2010,b; Voytek et al., 2010). A recent study reported a framework for detecting genuine CFC in human resting‐state using MEG in source reconstructed activity (Siebenhühner et al., 2020). Their research focused on n:m phase‐to‐phase and phase‐to‐amplitude interactions supporting the evidence of those two types of phase‐based CFC in human brain activity.

A well‐known estimator for quantifying AAC is the correlation of the envelope extracted after applying Hilbert transform on the bandpass filter time series. n:m PPC can be estimated for specific ratios of n:m factors after first extracting phase time series with the Hilbert transform with PLV or iPLV estimators. PAC CFC can be analyzed in two scenarios:

(a) bandpass filtering of time series in low and high frequencies, (b) extracting the phase and the envelope of the amplitude of low and high frequencies via Hilbert transform, (c) adopting a proper estimator for the quantification of PAC using the phase of the slow oscillation and the envelope of the high frequency (Bruns et al., 2004; Canolty et al., 2006; Penny et al., 2008; Tort et al., 2008) or extracting the phase from the envelope of the high frequency and adopting PLV (Lachaux et al., 1999), iPLV (Dimitriadis et al., 2015).
(a) bandpass filtering of time series in low and high frequencies, (b) extracting the phase and the envelope of the amplitude of low and high frequencies via Hilbert transform, (c) bandpass filtering the envelope of the high frequency within the frequency range of the slow frequency, (d) extracting the envelope of the slow oscillation within the high frequency via Hilbert transform, (e) adopting a proper estimator for the quantification of PAC (Bruns et al., 2004; Canolty et al., 2006; Penny et al., 2008; Tort et al., 2008), or extracting the phase from the envelope of the high frequency and adopting PLV (Lachaux et al.,1999), iPLV (Dimitriadis et al., 2015).

Our module includes all the CFC types and estimators mentioned above, with the primary goal to provide a common framework for exploratory analysis for neuroscientists. In a future version of the module, we will implement a new statistical framework for detecting true CFC and simultaneously PAC—AAC (Nadalin et al., 2019).

## 3 FROM STATIC TO DYNAMIC CONNECTIVITY

Representing brain connectivity as a graph structure, through the lens of graph theory, is only a natural outcome. Graph theory provides a robust mathematical framework to analyze the spatio‐temporal aspects of brain connectivity.

In general, a graph can be directed or undirected, weighted or unweighted (binary). A complex brain network can be interpreted as a collection of nodes, the brain regions and edges, the functional or structural connectivity between these nodes.

The definition of the nodes is related to the definition of the regions of interest. Depending on the study, the nodes may be as simple as the individual voxels themselves or, in some instances, the result of a parcellation pipeline (automatic or by the intervention of an expert). As we have already discussed in short, the edges are determined by measuring the interaction between the nodes. However, this further depends on the kind of complex network. In the case of structural networks, the edges represent physical, cordial interconnections. Moreover, in functional networks, there is an array of methods to choose from and build the statistical dependence among brain regions. The one commonly used is Pearson's correlation. Finally, effective connectivity concentrates on the direction of those interactions.

**FIGURE 1:**
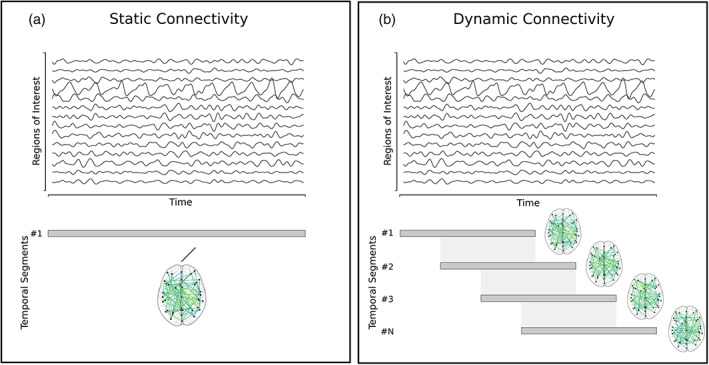
The two aspects of brain connectivity. (a) Static connectivity; all the samples are considered to estimate the interactions between the ROIs, and (b) a sliding and overlapping temporal window is used over the time series and ROIs. The connectivity is estimated for every temporal segment between the ROIs. The number of produced connectivity matrices depends on the size of the window and the overlap parameters

It is common to approach structural, effective or functional connectivity through computation of statistical inter‐dependencies among all brain regions, capturing the whole temporal dynamics into one single graph structure (Figure 1a).

The transition to dynamic connectivity can be as simple as segmenting the original time series according to a strategy and estimating the connectivity within each of these temporal partitions. The most well‐used method is the one of a temporal (sliding) window of length l, being shifted T number of samples. This yields multiple connectivity graphs capturing the connectivity evolution over the temporal intervals (Figure 1b).

There are a few considerations to decide upon when opting‐in a dynamic functional connectivity approach. In the case of the sliding window, the most apparent of them are the parameters of the sliding window, the width, the number of samples to use, and the stepping size; how many samples the window will slide over the time series. Both parameters affect the captured dynamics. A good rule of the thumb would be to consider the sampling frequency (fs) in the EEG/MEG modalities and the repetition time (TR) in the context of fMRI.

Our module has been developed around dynamic connectivity and provides implementations to both the classical method of the sliding window and the time‐varying framework. In the previous paragraphs, we discussed the method of the sliding window.

The other method that we provide in the module is the time‐varying functional connectivity graphs (TVFCGs) (Dimitriadis et al., 2010; Astolfi et al., 2008). This framework introduces the idea of processing overlapping segments of neuroelectric signals by defining a frequency‐dependent time window (cycle criterion, CC) in which the synchronisation is estimated. It regulates the amount of the oscillation cycles that will be considered in measuring the phase synchrony. A time‐window with width twice the lower period (CC=2.0) (Cohen, 2008) was initially proposed in the literature. On the other hand, TVFCGs, consider the given lower frequency that corresponds to the possibly synchronised oscillations of each brain rhythm and the sampling frequency. The overlapping is determined by an arbitrary step parameter. Given some neurophysiological recording data $X^{m\times n}$ (where m denotes the number of ROIs and n the number of samples in the neural time series), a frequency range with $F_{\mathrm{up}}$ and $F_{\mathrm{lo}}$ the upper and lower limits, fs the sampling frequency, step the stepping, and CC the cycle criterion. The length of the sliding window is given by $\frac{\mathrm{CC}}{F_{\mathrm{lo}}}\mathrm{fs}$ and the whole framework results in $\frac{n}{\text{step}}$ adjacency matrices. Figure 1b illustrates how TVFCG is constructed by sampling the functional recordings via the sliding‐window method.

### 3.1 Statistical—Topological filtering

Both, static and dynamic approaches, produce fully connected graphs—no matter the configuration (i.e., modality, connectivity estimator)—, potentially comprised of numerous spurious connections. It is a standard method to employ statistical methods to filter out these spurious connections (Aru et al., 2015). In the literature, quite a few approaches have been proposed. The most common one is that of surrogate analysis, according to which randomised connections are generated based on the original ones' profiles and a p‐value is assigned. Then, these p‐values are trimmed (thresholded) according to some criterion, such as false‐discovery rate (FDR) to control for type I errors. Numerous topological filtering schemes have been proposed so far spanning from arbitrary absolute thresholding up to data‐driven orthogonal spanning trees (OMSTs) (Dimitriadis and Salis, 2017; Dimitriadis et al., 2017).

#### 3.1.1 Surrogate analysis of functional brain networks

In order to detect true functional couplings at resting‐state or during cognitive tasks, it is essential to design proper surrogates of the original recordings. Then, one should reestimate the functional coupling with the adopted functional connectivity estimator between pairs of surrogates and build a distribution of surrogate functional connectivity values. Parametric and non‐parametric approaches can compare the original functional connectivity strength between every pair of time‐series or brain areas (atlas) to the surrogate values by assigning a p‐value to the observed coupling strength. Then, at network level, one can adjust for multiple comparisons using false discovery rate at a specific q‐value.

In brain connectivity analysis, we adopted three basic surrogates' types for testing phase synchronisation and coupling between oscillators using various measures, such as PLV, PLI, iPLV, mutual information (MI) and so forth. The following surrogate types were proposed for testing non‐linearity in data: Fourier transform (FT), amplitude adjusted Fourier transform (AAFT), and iterative amplitude adjusted Fourier transform (IAAFT) surrogates. FT, AAFT and IAAFT have been extensively studied in many applications (Theiler et al., 1992; Schreiber and Schmitz, 2000).

In a multi‐trial paradigm, Lachaux et al., (1999) suggested a trial‐based surrogates' analysis. For every pair of time series reflecting the activity of two brain areas, we can shuffle the order of the trials of the second brain activity and estimate the trial‐based surrogate functional coupling strength. Then, the procedure of correcting the observed coupling strengths is the same as the one aforementioned.

A suitable surrogate construction is debatable for functional interactions within‐the‐same‐frequency and CFC. An optimal surrogate algorithm tailored to CFC should remove cyclo‐stationaries related to the true CFC effect while keeping non‐linearities and non‐stationarities of the original data (Aru et al., 2015). This is an optimal scenario for surrogate construction which removes the effect of interest, but some surrogates algorithms are more conservative. In experimental paradigms with repetitive events, for instance, in events/trials that are locked to an external stimulus shuffling the whole phase or amplitude time series between events/trials is the most logical approach. However, an event‐related potential means that some pairs of frequency components will be locked simultaneously to the stimulus and between themselves. Shuffling trials/events cannot detect the source of modulation, and it is one of the most significant drawbacks of this method. Another surrogate approach used in many studies is the phase scrambling (shuffling) across experimental time. This approach returns stationary surrogates by removing specific and non‐specific non‐stationarities to the physiological mechanism and produces measures CFC (Nakamura et al., 2006). Such approaches that introduce surrogate‐based false‐positive CFC in the original data do not preserve the non‐stationarity profile of the underlying data not directly linked to the physiological nature of the production of CFC. An additional surrogate method suggested the block resampling a continuous time series was cut at several points, and the resulting temporal segments/blocks permuted randomly (Canolty et al., 2006). This method, again, suffers from the issue as Nakamura et al., (2006). Another approach could be to cut the original time series at a single point—located at a random location or over the middle of the time series—and exchange the two resulting temporal segments/blocks. Repetition of this procedure could produce a set of surrogates that would minimise the distortion of the original phase/amplitude dynamics. This surrogate type could be used in the three basic CFC types: phase‐to‐phase, phase‐to‐amplitude and amplitude‐to‐amplitude. This conservative surrogate construction approach could minimise the number of false‐positive CFC.

A recent statistical framework supports the detection of true CFC by considering both PAC – AAC simultaneously (Nadalin et al., 2019). This method will be implemented in an upcoming version of our module. This statistical framework for CFC should be further tested against the surrogate null models in multiple experimental paradigms and modalities.

We have proposed an integrated dynamic functional brain graph that encapsulates both the dominant coupling mode, either within‐frequency or CFC (DoCM model), and their strength (Dimitriadis et al., 2017, 2018). This integrated temporal network informs us how different brain systems communicated within and between each other across experimental time. The semantic information of dominant coupling modes encapsulates enriched information that can be summarised by the probability distributions of dominant coupling modes across brain structure and experimental time. Flexibility and complexity indexes can also be estimated over the symbolic time series that encode the dominant coupling modes per pair of brain areas across experimental time (Dimitriadis et al., 2017, 2018).

#### 3.1.2 Topological filtering of functional brain networks

Topological (or spatial) methods go beyond the traditional statistical approaches and define operations that are being applied on the connectivity graphs by considering the structure of the graph, locally or globally. Topological thresholding can be applied subsequently after statistical filtering to highlight essential network structures stemming from neuroscience (Bullmore and Bassett, 2011; Van Wijk et al., 2010; Fallani et al., 2017). The most common method is to filter out the edges based on an arbitrary value criterion. For example, given the absolute values of a graph, one can filter out those below a threshold (i.e., 0.5); based on upper‐density limits, keeping only the strongest 10% of the total connections; or retaining as many as connections needed for the mean degree of the graph to reach a minimum threshold (Dimitriadis et al., 2010).

**FIGURE 2:**
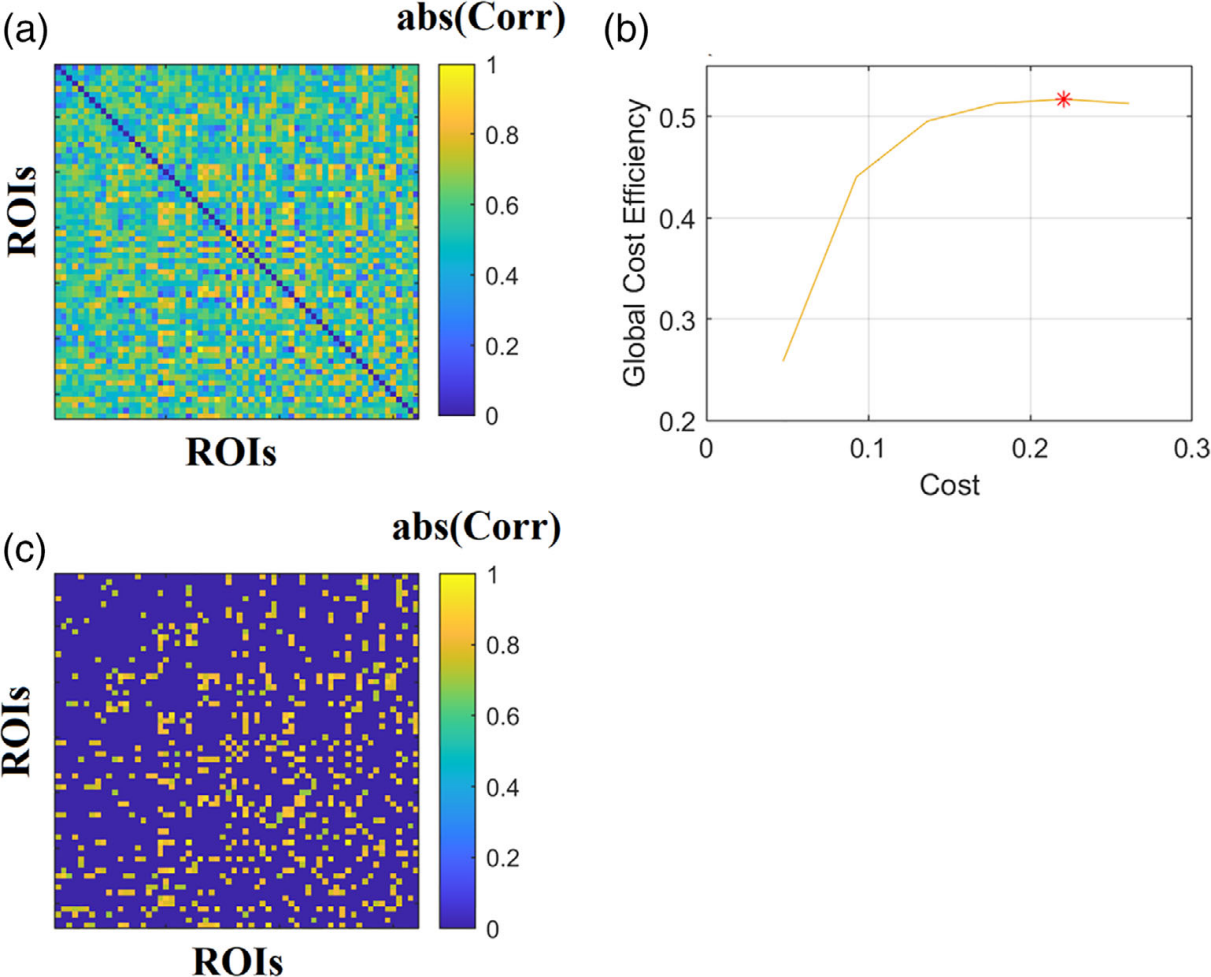
Data‐driven topological filtering of a functional brain network. (a) A fully‐weighted correlation map derived from 64 BOLD time series of a resting‐state condition. (b) Maximisation of global cost‐efficiency (global cost‐efficiency—cost) parameter over iterative orthogonal minimum spanning trees tills it gets its maximum value. (c) The threshold connected functional brain network as a sparser version of the original fully‐weighted brain network showed in (a)

In the literature, these thresholding schemes have been studied and compared with each other regarding their possible effect on clinical connectomic biomarkers (van den Heuvel et al., 2017) and their stability (Garrison et al., 2015) between groups. These crucial findings make up a strong argument for the need for data‐driven methods. Stemming from graph theory, Minimum Spanning Trees (MST) and Orthogonal Minimum Spanning Trees (OMST) (Dimitriadis et al., 2017) are unbiased, assumption‐free methods that identify significant links within a weighted graph, diminishing the need for statistical methods. MST produces a graph that connects N nodes through N−1 edges by minimising the total cost of information flow and without introducing cycles. An essential effect of MST is the sparsity of the resulting trees that have been deemed unreliable for discrimination between two (Antonakakis et al., 2016; Dimitriadis et al., 2015) or more groups (Khazaee et al., 2016). OMSTs address this issue through an iterative optimisation procedure that computes an MST and estimates the Global Cost Efficiency. The objective function is given by: $J_{\mathrm{GCE}}^{\text{OMSTs}}=\mathrm{GE}-\text{cost}$, where GE denotes the Global Efficiency of the network, a measure (as its name imply) of efficient information exchange. The cost is the ratio of the sum of an MST's weights over the total sum of the initial, fully weighted graph. The superiority of OMST over several conventional filtering schemes has been recently demonstrated in both EEG and fMRI modalities (Dimitriadis et al., 2017, 2018). Figure 2a illustrates a Pearson correlation brain network from a healthy control male subject of age 8 using BOLD activity from a resting‐state condition. The atlas used was based on bootstrap analysis of stable clusters (BASC) (Bellec et al., 2010) with 64 ROIs leading to a 64×64 fully‐weighted brain network. Applying the OMST data‐driven topological filtering over this functional brain network, we get a threshold brain network (Figure 2c) where its economical wiring is at the maximum (Figure 2b). OMST stops when global cost efficiency—cost versus cost gets a global maximum peak.

### 3.2 Network metrics time series

**FIGURE 3:**
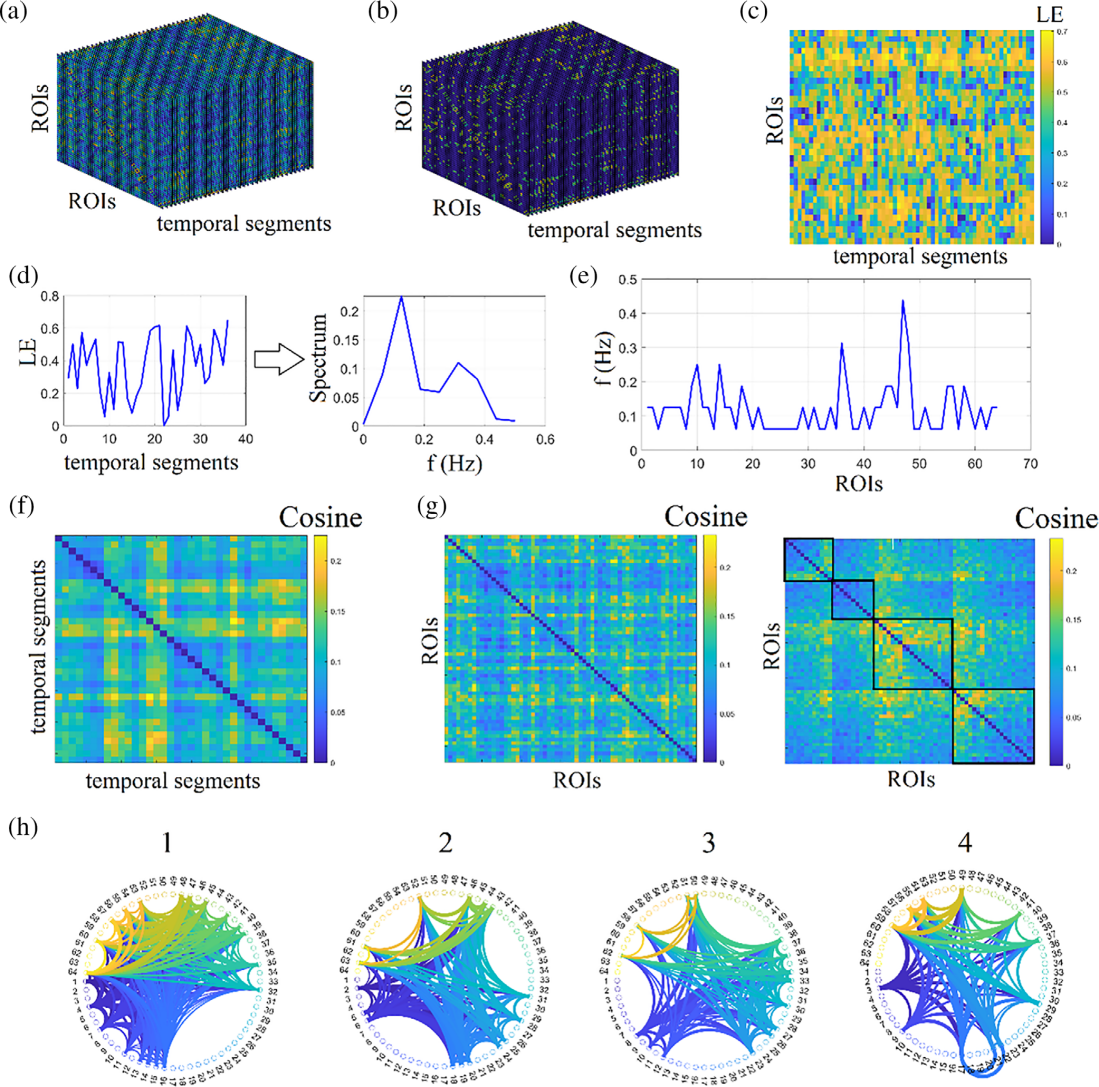
Network metrics time series of functional brain networks. (a) Original fully‐weighted dynamic functional connectivity brain graph. (b) Statistically and topologically filtering of dynamic functional connectivity brain graph. (c) Estimation of nodal local efficiency (LE) across time and ROIs producing the network metric time series. (d) An example of a nodal local efficiency and its spectrogram. (e) The characteristic dominant frequency of nodal local efficiency. (f) Cosine similarity matrix between every temporal pair of networks' nodal local efficiency. The higher the cosine value, the more similar are the temporal local efficiency nodal profiles. (g) Cosine similarity matrix between every pair of ROIs using their nodal local efficiency and its reordering according to the graph‐partition clustering algorithm to reveal the four nodal communities. (h) The demonstration of the four communities detected in G via a circular layout of the 64 ROIs

After the construction of static (structural or functional) or dynamic functional connectivity patterns, the next natural step is the estimation of global (whole network) or nodal (per node) network metrics. These network metrics—adopted from social network analysis—can be classified in those that detect functional integration and segregation, quantify the centrality or hubness of brain areas, characterising the shortest pathways among brain areas. The application of network metrics over a dynamic functional connectivity brain network can result in network metric time series (NMTS) that describes a network metric's temporal evolution across experimental time (Dimitriadis et al., 2010). Figure 3a shows the original temporal network constructed from fMRI resting‐state activity from a healthy control male of age 8 with 200 samples. We adopted a sliding‐window approach with a width of 20 TR and a step of 5, leading to 36 temporal segments and the PLV estimator. Then, the temporal network was statistically filtered with 1,000 surrogates using the single‐point cut criterion of every ROI based time series, producing two temporal segments that exchanged their order. We set the FDR criterion to 0.01. For topologically filtering, we employed OMST (Figure 3b). Nodal Local Efficiency (LE) was estimated per ROI and temporal segment, producing the image illustrated in Figure 3c based on NMTS^LE^. These NMTS can also be used to detect brain states (see next section; Dimitriadis et al., 2015). An exciting feature that can be extracted from these NMTS is its characteristic spectrogram using a fast Fourier transform (Figure 3d). Taking the dominant frequency from every ROI based NMTS, we can get the dominant oscillatory profile of every brain area related to how fast functional segregation changes over experimental time (Figure 3e).

NMTS is a matrix of size ROIs×temporal segments. This matrix can be used in two complementary scenarios. Firstly, identifying network similarity across an experimental time where high similarities can be interpreted as time instances with similar functional segregation profile. Secondly, for detecting groups of edges with a similar co‐fluctuation pattern of functional segregation. A cosine similarity matrix of size temporal segments×temporal segments has been estimated for examining network similarity across time instances using 36 NMTS row‐vectors (Figure 3f). Higher/lower values are related to transient stability/instability of NMTS^LE^. Secondly, to detect nodes communities, a cosine similarity matrix has been designed using the 64 NMTS column‐vectors (Figure 3g), expressing each one the temporal evolution of LE (Figure 3e). This similarity matrix was fed to a graph‐partition algorithm producing four clusters of nodes demonstrating in Figure 3h.

Brain connectivity toolbox (BCT) provides an extensive list of network metrics in many programming languages, including Python (Rubinov and Sporns, 2010). Our contribution to this topic is a set of network metrics tailored to analyzing the temporal and spatial (structural) dimension of networks under community detection, network analysis and their significance. This dynamic network sub‐module involves the multiscale and multiplex community detection algorithm (Mucha et al., 2010), community laterality that quantifies the extent in which a community is localised to the left or right hemisphere or in targeted sets of brain areas like the default mode network (Doron et al., 2012), the radius and diameter of a temporal community, the flexibility index that quantifies how many times a node changes community assignment between consecutive temporal segments (Bassett et al., 2011), cohesiveness strength which counts if two or more nodes change community assignment across experimental time together in conjunction to flexibility index (Telesford et al., 2017) and an inter‐slice γ coupling that quantifies how the strength of every node changes across time between consecutive temporal segments (Guillon et al., 2017). Temporal centrality, the shortest path length and the related small‐worldness index are also part of this sub‐module. Significance levels of these network metrics and the ones closely linked to temporal community assignment demand proper surrogate null models (see next section).

### 3.3 Surrogate null models for temporal networks

While the estimation of network metrics in static and temporal networks offer important information about the studied network structure, it is straightforward to assess if the observed network architecture deviates from statistical surrogate null models. For static graphs, well‐known null models include the Erdös‐Rényi random graph model (Erdos and Rényi, 1960), the ring lattice (Watts and Strogatz, 1998), and the configuration model (Newman, 2003), a random graph model that preserves the degree distribution and the connectedness of the network (Maslov and Sneppen, 2002).

**FIGURE 4:**
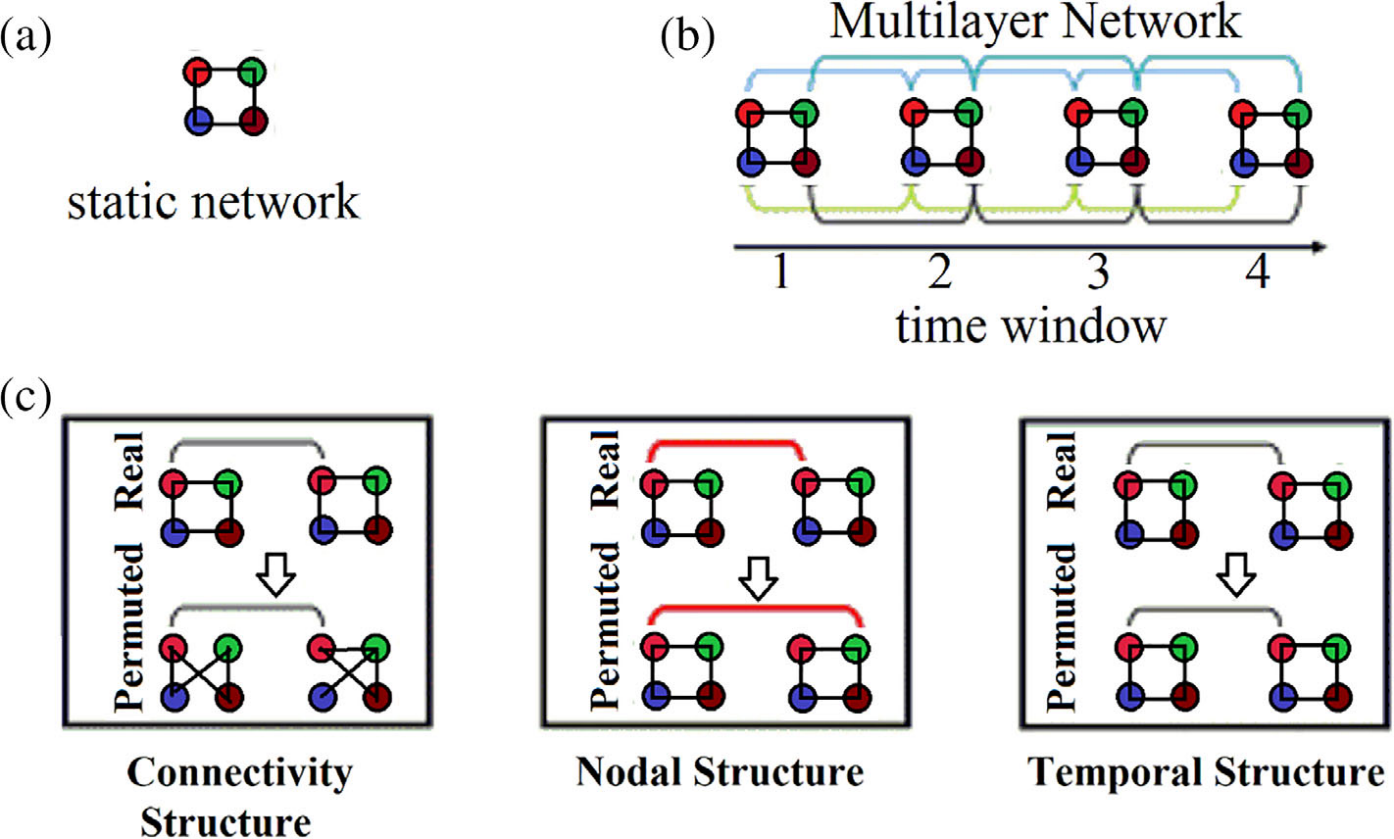
Temporal dynamics of modular architecture. (a) A static network toy example composed of four nodes. (b) A Multilayer network toy example composed of four time windows which are linked in pairs by connecting homologues nodes between adjacent time windows. (c) A statistical framework tailored to temporal networks that can be classified into a connectional null model (Top), a nodal null model (Middle), and a temporal null model (Bottom). These surrogate null models randomly permute the intra‐network links, internet‐work links, and time‐windows, of the original real temporal network. We show all the types of randomised links in red

The design of proper surrogate null models for static graphs is straightforward. However, in temporal networks, we must consider both edge weights between pairs of nodes at every time instance (intra‐network links), edge weights between pairs of nodes across temporal segments (inter‐network links) and temporal segments (time windows). All these alternative scenarios can be adopted one by one or simultaneously, producing an adequate number of null models (e.g., 1,000) and comparing the surrogate modularity, size—number of temporal modules and other temporal network metrics with the original ones (Figure 4). In fully‐weighted temporal networks, intra‐network links will exchange their functional strength and not their connectivity profile. We suggest that researchers should explore the three different surrogate null models independently and simultaneously.

## 4 FROM DYNAMIC FUNCTIONAL CONNECTIVITY GRAPHS TO BRAIN STATES

The first mining approach of spatiotemporal electroencephalographic (EEG) activity has been introduced by Lehmann et al., (1987). Lehmann's microstates (Lehmann, 1990; Lehmann et al. 1987, 2010) summarise quasi‐stable scalp EEG potential maps lasting from tens to hundreds of milliseconds. Brain EEG activity revisits these microstates supporting their complex behaviour. EEG microstates focused on the analysis of the EEG sensor or virtual sensor broadband activity.

Motivated by Lehmann's approach, we introduced a framework for the analysis of functional connectivity brain patterns (Dimitriadis et al. 2013, 2015, 2017, 2019). These newly represented microstates, called functional connectivity microstates (FCμstates), proved valuable descriptors of whole‐brain inter‐areal functional synchronisation during ERP responses (Dimitriadis et al., 2013), during cognition (Dimitriadis et al., 2015) and at resting‐state (Dimitriadis et al., 2017). This approach proved valuable for designing a reliable connectomic biomarker tailored to mild cognitive impairment (Dimitriadis et al., 2019).

Simultaneously, a pioneering study introduced brain states' notion in functional magnetic resonance imaging (fMRI) using an extensive study and adopting the k‐means clustering algorithm (Allen et al., 2014). Since then, many methods have been proposed to mine temporal networks that lead to brain states and their temporal evolution following a whole‐brain procedure (Cabral et al., 2017; Deco et al., 2019). A study similar to Lehmann's approach adopted Hidden Markov modelling to reveal the transient non‐random behaviour of brain activity using fMRI resting‐state recordings (Vidaurre et al., 2017).

The construction of functional connectivity brain networks can be realised via the adaptation of the sliding‐window.

### 4.1 Mining Time‐Resolved functional brain networks

It is often required to identify recurrent patterns of functional connectivity (FC) across time, subjects and populations. Given a data set of DFCGs, a typical data‐driven (Allen et al., 2014) framework includes the k‐means clustering algorithm's employment (Lloyd, 1982) to mine for common patterns. Then, the original DFCGs are reconstructed using these ‘k‐learned’ prototypes, based on some similarity metric (i.e., Euclidean distance, Pearson's r correlation). The number of k brain states is usually decided based on some secondary estimates, such as the elbow rule with the Silhouette index, mean square error (MSE), distortion, etc. However, these estimates heavily rely on the users' expertise (Vergara et al., 2019).

The notion of these ‘FC states’ (brain states) is usually compared with that of the EEG microstates (Lehmann 1990; Pascual‐Marqui et al. 1995), according to which the brain topography remains quasi‐stable across a period of time. The focus of the research community on brain states have spiralled out a vast number of research studies. In the original article (Allen et al., 2014), the authors observed the mined brain states' replicability across the cohort at hand using the k‐means clustering algorithm. In the same spirit, Abrol and his team identified a small number of repeatable brain states across 28 independent groups with 250 subjects each (more than 7,000 fMRI data sets) (Abrol et al., 2016, 2017).

K‐means is the most popular clustering algorithm in the literature. However, by default, it is a non‐deterministic algorithm which practically means that we can get a different number of clusters (here brain states), at every run. This drawback is explained by its gradient descent nature which characterises its sensitivity to the initial assignment of cluster centres. Many different approaches have been introduced so far to address this issue, with the primary aim to transform k‐means in a deterministic algorithm. Tens of studies adopted the k‐means algorithm to detect brain states taking advantage of the first study by Allen and her colleagues (Allen et al., 2014), which introduced this approach. We recommend to the researchers to pay attention to the usage of k‐means in general and specifically for the detection of brain states. A solution to this issue is to run the k‐means algorithm multiple times, summarise the results into a consensus clustering and then repeat the clustering with a deterministic graph‐based approach or adopt, by the beginning of the analysis. K‐means++ could be an alternative solution, because it provides clusters with more independence from the random initialisation of centroids than the original k‐means (Arthur and Vassilvitskii, 2007).

We, as a group, introduced the detection of brain states which is a summarisation of functional connectivity patterns called FCμstates, by adopting neural‐gas (NGAS) algorithm (Martinetz et al., 1993). The neural‐gas algorithm is a non‐deterministic algorithm by default, but we transformed it in a deterministic by small adjustments. It proved its capability to describe temporal networks into a small repertoire of FCμstates in multiple data sets and experiments (Dimitriadis et al., 2013, 2015, 2017, 2019). In a recent study, using magnetoencephalographic resting‐state recordings from multiple scans, we proved the replicability of brain states across brain frequencies (Dimitriadis et al., 2018).

In a recent EEG music study (Marimpis et al., 2016), we further enhanced the descriptive power of the extracted FCμstates by pre‐learning the manifold of dynamic functional connectivity patterns with non‐negative matrix factorisation (NNMF) (Lee and Sung, 1999). It was the very first time that NNMF was applied to dynamic functional connectivity patterns. We employed NNMF to approximate the original dynamic functional connectivity patterns via a two‐dimensional vectors approximation which then fed to NGAS to detect a k number of brain states (prototypes; FCμstates) (Marimpis et al., 2016). This approach proved valuable for the design of chronnectomic brain age index (Dimitriadis et al., 2017). NNMF has proved a valuable tool for identifying connectivity patterns related to autism (Zhou et al., 2020).

Alternative algorithmic scenarios of studying dynamic functional connectivity patterns are the usage of principal component analysis (PCA) and singular value decomposition (SVD) (Leonardi et al., 2013). Both PCA and SVD revealed the so‐called eigenconnectivity prototypes accompanying their time‐dependent eigenconnectivity profile.

The above usage of the algorithms is the conversion of dynamic functional connectivity brain networks into a discrete set of symbols, one per FCμstate, leading to a symbolic time series. This time series describes the temporal evolution and transition of brain states across experimental time in a similar temporal detail as the original dynamic functional connectivity brain network.

**FIGURE 5:**
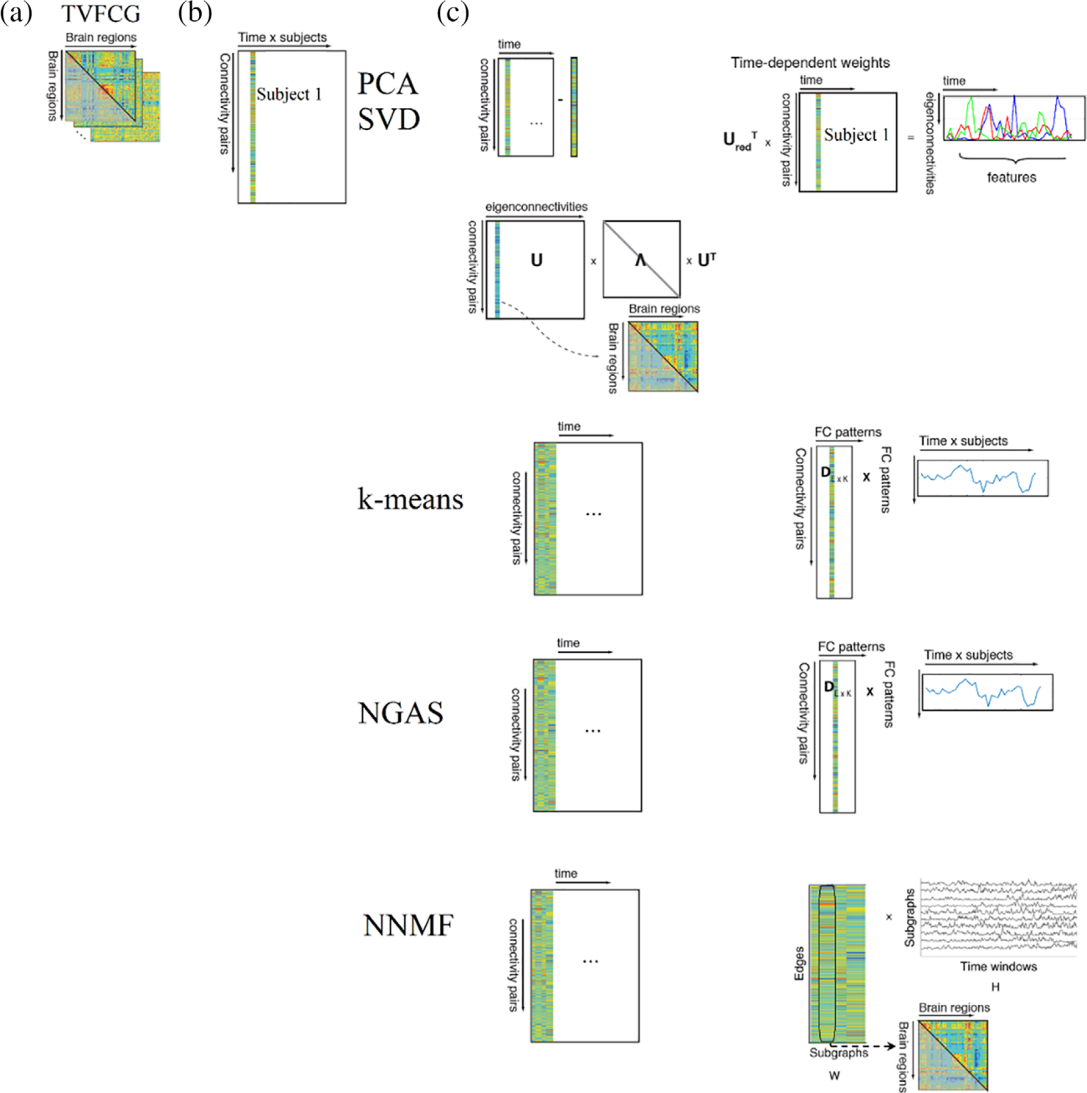
Flowchart of the analytic pipeline of time‐varying functional brain networks. (a) Construction of time‐varying functional connectivity graph (TVFCG). (b) Vectorisation of TVFCG in the dimension of connectivity pairs×time×subjects. (c) PCA/SVD demands a demeaning process that is performed by subtracting the static functional connectivity graph of every subject by every time‐dependent functional connectivity graph. Then, we can project the feature matrix to a new feature space that includes the eigenonnectivities and their temporal weights. K‐means and Neural Gas decompose the vectorised TVFCG leading to FC patterns (brain states) with the related symbolic time series that expresses the temporal evolution of FC patterns across time and subject. Non‐negative matrix factorisation decomposes the vectorised TVFCG leading to two‐rank approximation matrices. The first axis refers to FC patterns (brain states) and the second one, to the time‐dependent magnitude that expresses the weight of every FC pattern across experimental time

Figure 1b illustrates the construction of time‐dependent functional connectivity brain graphs and their modelling to brain states. Figure 5 shows how the algorithms of PCA, SVD, k‐means, NGAS and NNMF perform. Before processing any TVFCG (Figure 5a) with these algorithms, we must vectorise it. In the case of undirected TVFCG, the number of the first dimension equals the total number of pair‐wise connections: $N_{x}\frac{N-1}{2}$ where N denotes the number of ROIs (Figure 5b). The second dimension refers to the total number of subjects multiplying by the total number of time windows. Figure 5c illustrates how these algorithms process the vectorised TVFCG. PCA/SVD demands a demeaning process that practically is performed by subtracting the static functional connectivity graph of every subject from every time‐dependent functional connectivity graph. Then, we can project the feature matrix to a new feature space that includes the eigenonnectivities and their temporal weights. The outcome of PCA/SVD is a set of eigenconnectivities with the related time‐dependent magnitude that expresses the weight of every eigenconnectivity across experimental time (Leonardi et al., 2013). K‐means and NGAS decompose the vectorised TVFCG to a repertoire of FC patterns (brain states) with the related symbolic time series that expresses FC patterns' temporal evolution across time and subject. From this symbolic time series, numerous chronnectomics can be estimated (see Section 4.3). NNMF decomposes the vectorised TVFCG leading to two‐rank approximation matrices. The first refers to FC patterns (brain states), and the second to the time‐dependent magnitude that expresses every FC pattern's weight across experimental time. It is essential to state here that k‐means and NNMF lead to a different outcome at every run. We recommend running the algorithms multiple times and integrating them via a consensus approach.

### 4.2 Determining the number of brain states in dynamic functional brain connectivity using various cluster validity indexes

Using any type of the algorithms above (NGAS, PCA, NNMF, SVD) for detecting brain states from a dynamic functional brain connectivity graph demands the adaptation of a cluster validity index (CVI). It is common to use the Elbow‐Criterion, Silhouette index and the GAP‐Statistic. Ιn our module, we provide two indexes; the Davies‐Bouldin and Ray‐Turi index. The following will be included in an upcoming release: Calinski‐Harabasz, Silhouette, Wemmert‐Gancarski, Dunn, Log Det Ratio, Log SS Ratio, Scott‐Symons, SD Scat, Trace W. It is of paramount importance to validate brain states detection algorithms with NGAS, k‐means, PCA/SVD, NNMF and other methods in large data sets with repeated scans (Vergara et al., 2019, 2020).

### 4.3 Chronnectomics

Chronnectomics (where Chronos the greek word for time, and ‘omics’ describes the efficacy of objects being categorised based on common traits) is a relatively new class of neuroimaging term (Allen et al., 2014; Calhoun et al., 2014) that acts as the spatio‐temporal counterpart of connectomics. Genomics, metabolics and proteomics seek to study and understand both the healthy and diseased human organism. Similarly, chronnectomics are focused on the human brain. Chronnectomics integrate spatio‐temporal details pronounced from the distinct brain states, as extracted from a dynamic connectivity strategy. The outcome of k‐means and NGAS is a symbolic time series that describes brain states' temporal evolution.

**FIGURE 6:**
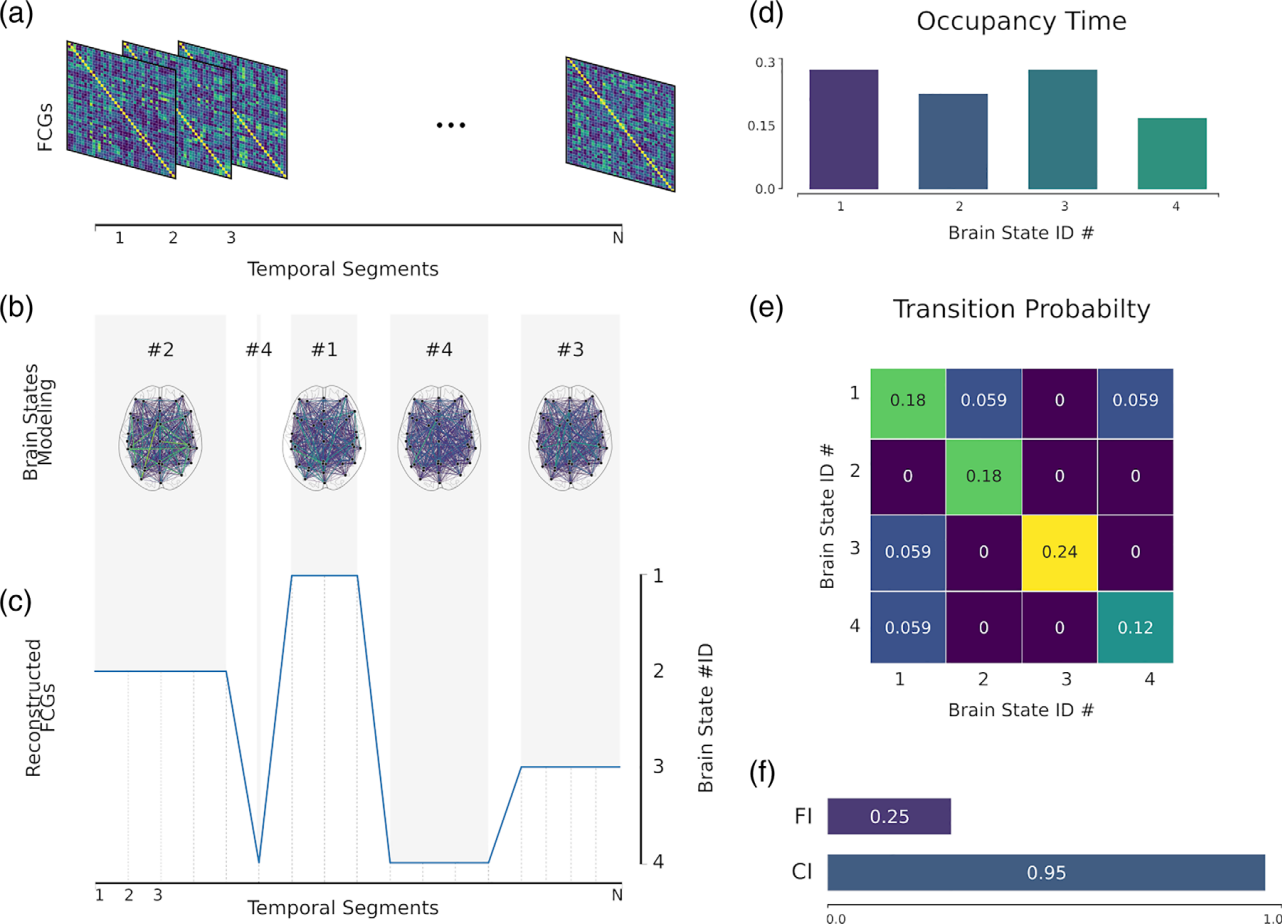
Illustration of a dynamic connectome pipeline. (a) The estimated dynamic connectivity, resulting in N functional connectivity graphs (FCGs). (b) Mined brain states; in this example four states. (c) Reconstruction of the original FCGs based on the mined brain states. (d) Occupancy time of each brain state; how time a brain state is active. (e) The transition probability of each brain state and between each state. (f) Flexibility index and complexity index for the symbolic time series

The symbolic time series extracted from the mining of temporal networks as introduced in the previous section and shown in Figure 6c using either k‐means or NGAS, numerous chronnectomic features can be defined. These chronnectomics are described below:

Flexibility index (FI) (Bassett et al., 2011, Dimitriadis et al., 2017; Dimitriadis, 2018) is the frequency of a node changing module allegiance; brain states' transition between consecutive temporal segments. The higher the number of changes, the larger the FI will be.

Given a symbolic time series, the flexibility index is estimated as follows:

(1) $$\mathrm{FI}=\frac{\text{number of transitions}}{\text{total symbols}-1}.$$

Occupancy time (OT) is the fraction of distinct symbols occurring in the observed symbolic time series. In the context of brain states (and dynamic functional connectivity specifically), for each brain state k, it is computed as

(2) $$\mathrm{OC}\left(k\right)=\frac{\text{frequency of}\;o\text{ccurance}}{\text{slides}}.$$

Dwell time (DT) measures when a brain state is active (highlighted) between consecutive temporal segments. The difference with the Occupancy Time is the search scope of the methods. DT captures the time that an individual spends in a given state; while OT measures the overall time spent in a given brain state.

Transition matrix (TM) tabulates every brain state's transition from one to another in a matrix form. One can convert this transition matrix into probabilities (transition probability matrix) by dividing the individual transitions by the sum of all observed transitions.

Complexity index (CI) (Janson et al., 2004; Dimitriadis, 2018) quantifies the ‘richness’ of symbolic time series. The index, specifically, indicates the frequency of repeated sub words (up to a specific length). It is to derive randomised versions of the original symbolic time series and estimate the index for each of them. Repeating this process 1,000 times, a distribution is generated that is used for the evaluation (i.e., consider the *SD*) and the normalisation of the original, resulting index.

Figure 6 illustrates an example of the majority of the chronnectomics above.

## 5 DIFFERENT FUNCTIONAL BRAIN NETWORKS REPRESENTATIONS

Numerous alternative representations of functional brain networks have been proposed during recent years, from original low‐order functional brain networks to high‐order and associated high‐order (Zhang et al., 2017). Multi‐layer functional brain networks have been used to express either temporal network (horizontal representation) with every time‐instant functional brain network‐referred to as a different layer (Mucha et al., 2010). Another representation is the integrated functional brain networks (vertical representation), with every layer referring to a distinct frequency‐dependent functional brain network (Dimitriadis et al., 2018). Finally, the edge‐to‐edge functional brain network deriving from a temporal network that encapsulates the co‐fluctuation functional temporal strength pattern between edges of pairs of brain areas.

### 5.1 Low‐order, high‐order and associated high‐order functional brain networks

**FIGURE 7:**
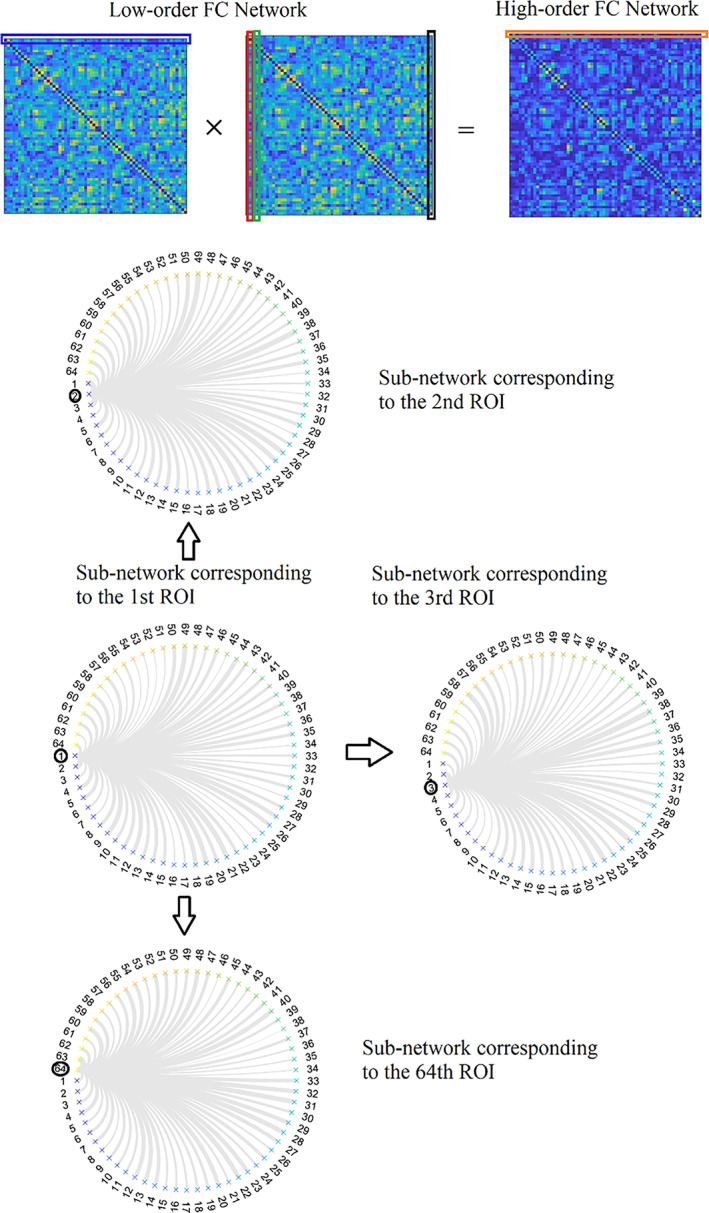
Illustration of high‐order FC network construction based on the topographical similarity between each pair of the rows of the first instance of a low‐order network with the columns of the second instance of a low‐order network. Circular graph layouts represent the rows or columns of a low‐order network, referring to a brain area's functional connectivity with the rest of the brain areas in a pair‐wise fashion

**FIGURE 8:**
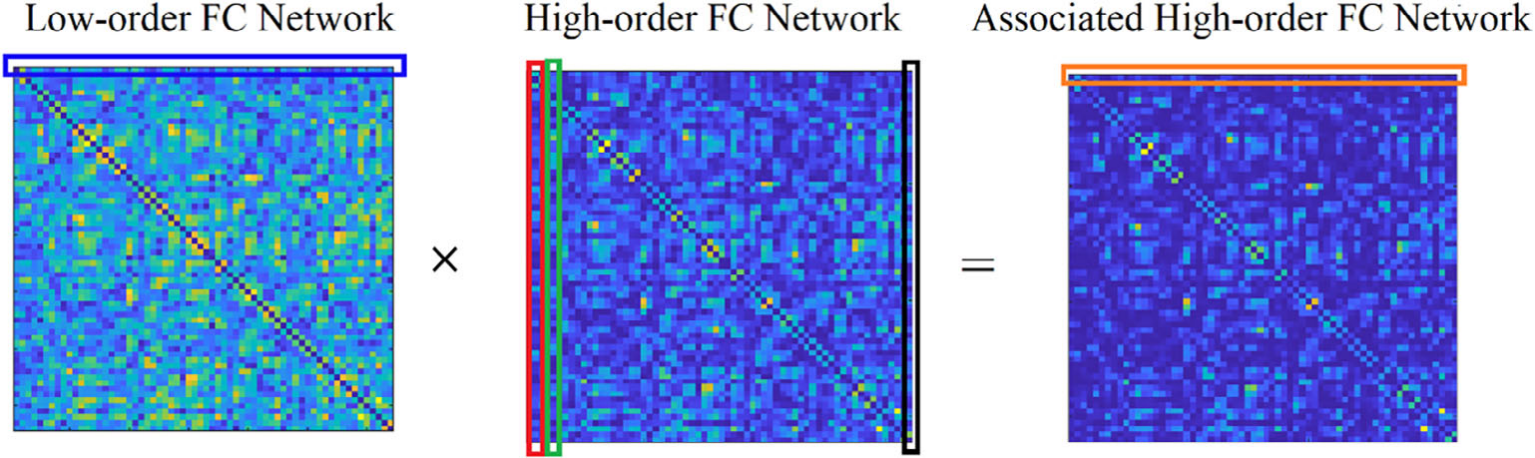
Illustration of associated high‐order FC network construction based on the topographical similarity between each pair of the rows of low‐order sub‐networks and columns of high‐order sub‐networks

A recent study introduced the notion of low‐order, high‐order and associated high‐order or hybrid high‐order functional brain networks (Zhang et al., 2017). Conventional functional brain networks are constructed by adopting a connectivity estimator and quantifying the temporal correlation between every pair of brain areas. This type of well‐known functional brain network is called low‐order (Figure 7). A high‐order functional connectivity graph (FCG) is constructed by taking the correlation between every pair of nodal correlation strength vector. Figure 7 illustrates how low‐order FCG constructs high‐order FCG. Every row from the first low‐order FCG is correlated with every column of the second low‐order FCG. Circular graph topological layouts illustrate the first row's functional strength from the first low‐order FCG and the second, third and last column from the second low‐order FCG. We used an example from a male of age nine from an fMRI resting‐state activity using BASC atlas with 64 ROIs. The low and high‐order FCG dimension is 64×64. The dimension of the rows and columns is a vector of 64, tabulating the functional strength values. The operation is a point‐wise of the first low‐order FCG with its inverted version. Following the same procedure between the resulting high‐order FCG and the low‐order FCG, one can estimate the associated high‐order FCG (Figure 8). Associated high‐order FCG are by default asymmetrical and could be treated as either a directed graph or undirected by taking the maximum value for every pair of nodes.

The whole operation can also be realised in a dynamic fashion by adopting the sliding‐window method. Topological filtering and network metric analysis can also be applied to the constructed high‐order and associated high‐order FCG as with the original low‐order FCG (Zhang et al., 2017). It is interesting, in the future, to explore these types of FCG in numerous experimental paradigms.

### 5.2 Multi‐layer networks

**FIGURE 9:**
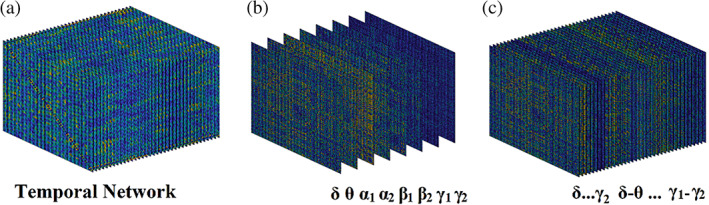
Illustration of different versions of a multi‐layer network. (a) A temporal network οf 36‐time instances. (b) A frequency‐dependent multi‐layer network where every layer refers to a functional brain network tailored to a frequency from δ to γ_2_. (c) A frequency‐dependent multi‐layer network where every layer refers to a functional brain network tailored to a within‐frequency coupling from δ to γ_2_ (8 layers) and also to every possible pair of cross‐frequency coupling between the eight frequencies (28 layers)

In recent years, the network neuroscience community introduced in the analysis of functional brain connectivity the notion of multi‐layer networks. The term multi‐layer network has been adopted to describe both temporal networks and multi‐frequency networks. In the first case, every layer refers to a time‐instant (Mucha et al., 2010). In the latter, every layer refers to a frequency‐dependent functional brain network (Guillon et al., 2017; Yu et al., 2017; Dimitriadis et al., 2018). Figure 9a shows a temporal multi‐layer network with 36 time‐instant functional brain networks. This temporal network is built from fMRI resting recordings from a healthy male of age 9. Figure 9b illustrates a multi‐layer multi‐frequency network where every layer is devoted to a frequency‐dependent functional brain network from δ up to γ_2_ (Guillon et al., 2017; Yu et al., 2017). Ιn a recent study, we first introduced a multi‐layer network that incorporates functional brain networks from both within‐frequency and cross‐frequency interactions between every pair of the studying frequencies, here δ to γ_2_ (Dimitriadis et al., 2018). Figure 9c is devoted to an integrated multi‐frequency network where every layer is devoted to a distinct coupling mode, 8 layers for within‐frequency coupling and 28 layers for CFCs. These networks (Figure 9b,c) were constructed from magnetoencephalographic resting‐state recordings from virtual time series located over the 90 ROIs according to the automated anatomical labelling atlas (AAL).

### 5.3 Edge‐to‐edge networks

**FIGURE 10:**
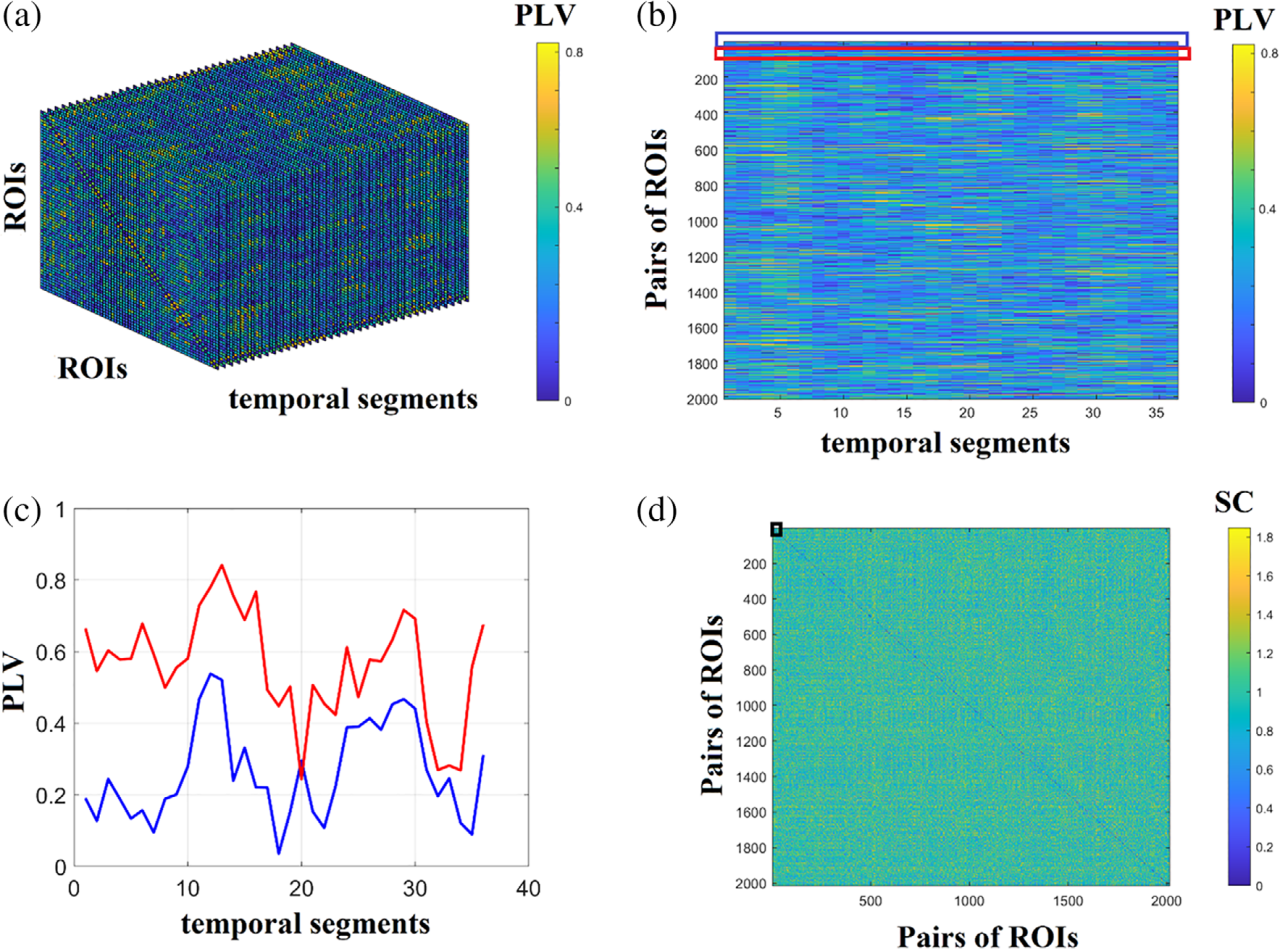
Schematic step by step construction of an edge‐to‐edge functional brain network. (a) An example of a 3D dynamic functional connectivity brain network. (b) A vectorised version of the 3D dynamic functional connectivity brain network into a 2D representation of temporal functional strength. (c) An example of temporal functional strengths for two pairs of pairs of ROIs $\left\{1-2,1-3.\right\}$. (d) The final edge‐to‐edge functional connectivity network estimated via spearman's correlation of every pair of temporal functional strength tabulated in the 2D matrix showed in B in total. Black box points the position of spearman correlation between the two temporal functional strength showed in C (PLV, phase locking value; SC, spearman's correlation)

An interesting representation of a dynamic functional connectivity graph is the edge‐to‐edge functional brain network. Figure 10a demonstrates the 3D dynamic functional network of dimensions temporal segments×ROIs×ROIs. Figure 10b is the transformation of the 3D dynamic functional network shown in Figure 10a to a 2D matrix of size possibleROIpairs×temporal segments. This matrix encapsulates the fluctuation of functional strength for every pair of ROIs. Figure 10c shows an example of temporal functional strength for pairs of ROIs $\left\{1-2,1-3\right\}$. By estimating the correlation between every possible pair of temporal functional strength time series, we build the edge‐to‐edge network presented in Figure 10d. The dimension of edge‐to‐edge functional brain network has dimensions of size possibleROIpairs×possibleROIpairs. The black box points to the spearman correlation between the two temporal functional strength shown in Figure 10c. If N is the number of ROIs, the possible pairs of ROIs equal to $N_{x}\frac{N-1}{2}$ for undirected functional brain networks and $N^{2}-N$ for directed functional brain networks.

### 5.4 Novel adjusted network metrics to different network representations

Trivial network metrics and transformations like topological filtering can be applied to low‐order, high‐order, associated high‐order and edge‐to‐edge functional brain networks. However, for multi‐layer networks, network metrics should be adjusted. Community detection of a multi‐layer temporal network demands an adjustment of famous graph partition algorithms like modularity (Mucha et al., 2010). Based on temporal community affiliations, various network metrics can be estimated. These metrics include *community laterality* that quantifies the extent to which a community is localised to the left or right hemisphere or in targeted sets of brain areas like the default mode network (Doron et al., 2012), the *radius and diameter of a temporal community*, the *flexibility index* that quantifies how many times a node changes community assignment between consecutive temporal segments (Bassett et al., 2011). Another one, the *cohesiveness strength*, counts if two or more nodes change community assignment across experimental time together in conjunction to flexibility index (Telesford et al., 2017) and an *inter‐slice γ coupling* that quantifies how the strength of every node changes across time between consecutive temporal segments (Guillon et al., 2017).

For multi‐layer frequency‐dependent functional brain networks, trivial network metrics should be adjusted properly. An example is the participation coefficient. The multi‐layer version of participation coefficient (MPC) quantifies the importance of every ROI across the different layers (Battiston et al., 2014). Brain ROIs with high *MPC* are characteristic central hubs of the ML‐FCG. MPC quantifies the importance of a node in a single‐layer or across layers. MPC tends to be 0 when an ROI has more connections within one layer, while it tends to 1 when an ROI distributes their connections across the layers. MPC has a meaning in topological filtered functional brain networks and not to fully‐weighted. A weighted version of MPC will be introduced in our module.

## 6 BRAIN NETWORK SIMILARITY

**FIGURE 11:**
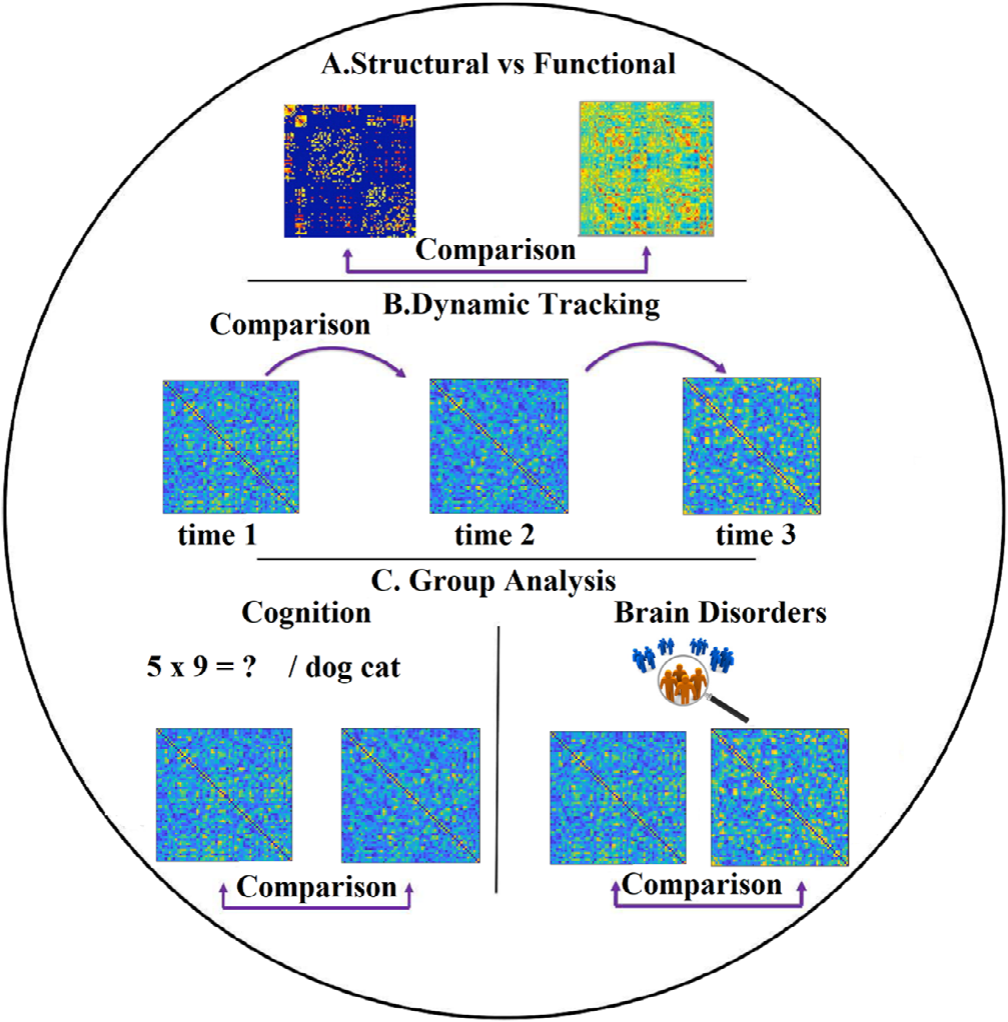
Applications of graph comparison in network neuroscience. (a) Comparison between structural and functional brain networks. (b) Tracking the dynamic (dis)similarity of a temporal network across time. (c) Comparison of sets of brain networks between two or more groups and conditions

Comparison of brain networks is an important and mandatory process in numerous network neuroscience studies. Necessary but not limited examples of those comparisons are: (a) the estimation of the (dis)similarity between structural and functional brain networks (Figure 11a), (b) tracking temporal similarity in a dynamic functional brain network between consecutive time window (Figure 11b). This analysis can also be used between a baseline static functional brain network and a dynamic functional brain network related to the active task and 3) the estimation of the dissimilarity between sets of brain networks related to normal and pathological groups or between two conditions in cognitive tasks (Figure 11c) (Dimitriadis et al., 2012, b; Avena‐Koenigsberger et al., 2018, Paban et al. 2019, Rizkallah et al. 2019).

Estimating (dis)similarity between networks in general, specifically for brain networks, is a complicated task based on the complex nature of the brain, which can be represented simultaneously across time, space, frequency, and modalities (structural, functional brain networks). Graph distance metrics can be classified based on the nature of the features extracted from brain networks: these features could be related to nodes, edges, network and spectrum level. In our previous study, we proposed a J‐index quantifying the dissimilarity of partition distance between pairs of communities between two cognitive tasks (Dimitriadis et al., 2012). Another study proposed a similar distance index quantifying the dissimilarity between sets of Laplacian eigenvalues on a node level (Shimada et al., 2016). Here, we will discuss two families of existing methods: (a) a graph theoretic approach which is based on the extraction of topological features from various types of sets of brain networks at network, nodal and edge level (Bassett and Sporns, 2017) (Bullmore and Sporns 2009, Zalesky, Fornito et al. 2010) and (b) *graph matching algorithms* including subgraphs isomorphism (Cordella et al. 2004), graph edit distance (Gao et al. 2010), and other approaches (Dimitriadis et al., 2012, b; Shimada et al. 2016, Schieber et al. 2017). Our module's current version provides a submodule with most of the graph distance metrics we discussed. Future releases will include methods to support subgraph isomorphism (Cordella et al. 2004).

### 6.1 Graph‐theoretic distance metrics

Comparison of graph‐theoretic metrics estimated on the network level can be realised via trivial statistical analysis (ANOVA, ANCOVA etc.). Similarly, for comparative analysis on the edge level, one can apply statistics for every pair of connections between two sets of functional or structural strength and then apply a multiple comparison correction approaches, like false discovery rate. However, this last approach presupposes that we deal with fully‐weighted brain networks, a solid and wrong assumption. Nodal network metrics produce a distribution of values equals to the size of the studying network. For a group or condition comparison, we get two or more sets of patterns/distributions. Every trace $\left\{X_{i}\right\},\left\{Y_{i}\right\}$ tabulates a matrix of dimension subjects×ROIs with the corresponding values to the adopted network metric. In the following equation, we express a J index used in our previous studies (Dimitriadis et al., 2012 b, 2013) that quantifies the dissimilarity ratio between every pair of inter‐set scatter and the two within‐set scatter.

(3) $$J=J\left(\left\{X_{i}\right\},\left\{Y_{j}\right\}\right)=\frac{IS_{\text{Cond}\;A↔\text{Cond}\;B}}{WS_{\text{Cond}\;A}+WS_{\text{Cond}\;B}}=\frac{1}{2}\frac{\sum_{i=1}^{N}\sum_{j=1}^{N}D\left(X_{i},Y_{j}\right)}{\sum_{i=1}^{N}\sum_{j>1}^{N}D\left(X_{i},X_{j}\right)+\sum_{i=1}^{N}\sum_{j=1}^{N}D\left(Y_{i},Y_{j}\right)}.$$

As a proper dissimilarity measure $D\left(\left\{X_{i}\right\},\left\{Y_{i}\right\}\right)$, we can adopt one of the many options provided in the following subsections. Dissimilarity measures should be tailored to the quantity that will be compared across groups or conditions. Below, we summarise sets of distance metrics that are implemented in our module.

#### 6.1.1 Partition distance

In our previous studies, we adopted the J index with a proper partition distance metric called Variation of Information (VI) (Dimitriadis et al., 2009, 2012). If our strategy is to compare graph communities between groups or conditions, we can adopt the J index with VI (Meila, 2007). In our module, we have implemented VI and normalised mutual information.

#### 6.1.2 Histogram distances

Adopting any network metric estimating on a node level produces a distribution of nodal network metrics per subject. As a proper distance metric to compare sets of histograms $\left\{X_{i}\right\},\left\{Y_{j}\right\}$ could be one of the following available: Kullback–Leibler Divergence, Jenson‐Shannon Divergence, Jeffrey Divergence, Chi‐Square, Kolmogorov–Smirnov, (Histogram) Intersection, (Histogram) Match, Quadratic form (Schauerte et al., 2012).

#### 6.1.3 Graph‐diffusion distance

The graph diffusion distance (GDD) metric measures the distance between two (positive) weighted graphs based on the Laplacian exponential diffusion kernel. The notion backing this metric is that two graphs are similar if they emit comparable patterns of information transmission. (Hammond et al., 2013). This GDD metric can be used to compare two structural brain networks, a structural brain network with every time‐instant temporal network and, at the same time, as a proper metric for comparing two sets of brain networks via J index.

#### 6.1.4 Spectral distance metrics

Given an undirected network, the Laplacian matrix L is computed as the difference between the degree (D) and adjacency matrix (A) accordingly: L=D−A. It maintains the same information as the adjacency matrix but has different, useful vital properties. Its spectrum, formed by its eigenvalues, can characterise a distinct neural network model from scale‐free and small‐world. The Laplacian spectrum of the normalised Laplacian matrix can inform us about the underlying brain network's basic topological properties. The first eigenvalues reflect the existence of communities with smaller first eigenvalues to support strong communities. Interestingly, the synchronizability index is defined as the ratio of the second smallest eigenvalue versus the largest eigenvalue. Peaks in the Laplacian spectrum indicate duplications or additions of characteristic motifs that arise from the network topology. The largest eigenvalue is an index of the bipartiteness level of the most bipartite subnetwork, linked to the odd cyclic motifs within a network (de Lange et al., 2014). In our module, we provide the following spectral distance metrics: (a) Ipsen‐Mikhailov distance (Donat and Holmes, 2018), (b) Spectral Euclidean Distance (Wilson and Zhu, 2008), (c) Spectral K distance (Pincombe, 2007), (d) Laplacian energy (Gutman and Zhou, 2006). This set of four spectral distance metrics implemented in our module can be used in conjunction with the J index.

### 6.2 Comparing temporal networks with structural brain network

**FIGURE 12:**
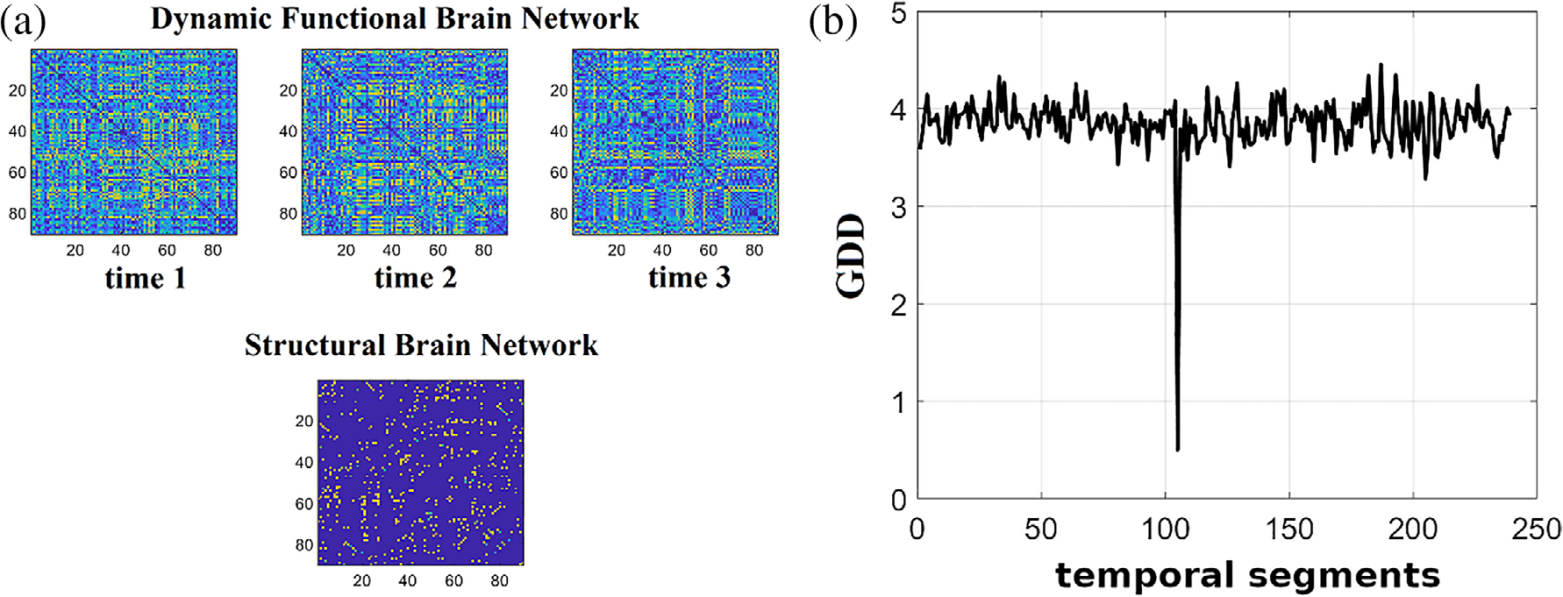
A graph distance topological similarity between a structural brain network and a dynamic functional brain network. (a) A structural brain network and multiple snapshots of a dynamic functional brain network. (b) Graph diffusion distance time series that represents the structural‐functional distance at every temporal segment. The peak around the temporal segment 105 depicts the remarkable similarity between the structural and functional brain networks

To demonstrate a way of quantifying similarities in a multimodal neuroimaging scenario, we used an integrated structural brain network produced as described in our study (Dimitriadis et al., 2017) and a dynamic functional brain graph produced using iPLV estimator from low—γ activity. Both types of brain networks were estimated using the AAL atlas of 90 ROIs (45 per hemisphere). Figure 12a shows the integrated structural brain network and a few snapshots from the dynamic functional brain network. Adopting GDD as a proper distance metric, we quantified their distance per temporal segment (Figure 12b). Fluctuations of topological similarities could be used for further comparisons across frequency bands, across subjects or by direct comparisons between groups or conditions.

## 7 GRAPH SIGNAL PROCESSING PROCESS ON FUNCTIONAL NEUROIMAGING BRAIN GRAPHS

**FIGURE 13:**
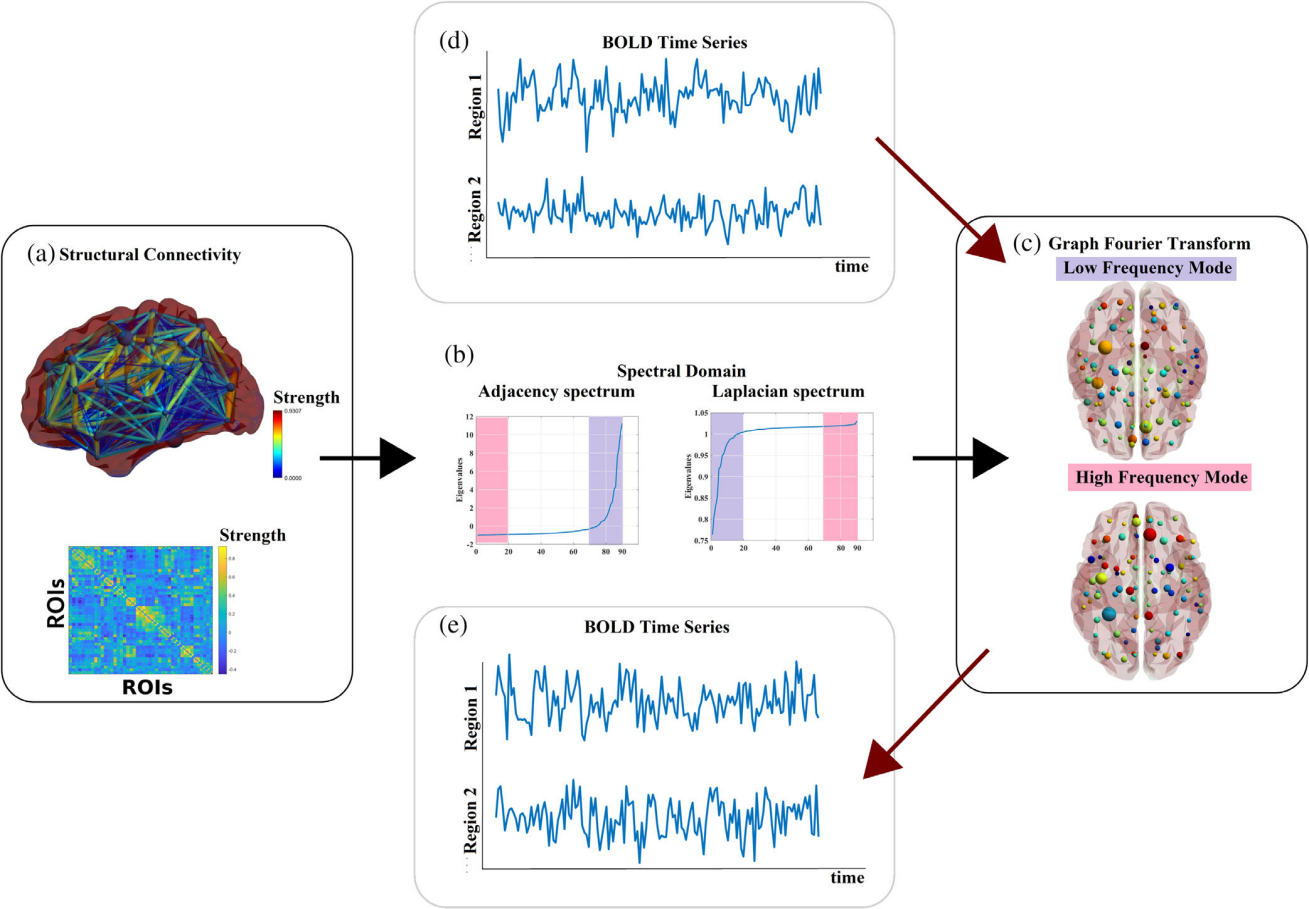
Graph signal processing for brain imaging. (a) Visualisation of a weighted structural connectivity graph derived from diffusion MRI in a sagittal brain view (top) and as an adjacency matrix of size ROIs×ROIs (90×90) where every edge (topography) or cell (adjacency) represents the strength of the structural connection. Here, we adopted a healthy subject of age 24. The matrix has been constructed as described in a recent study (Dimitriadis et al., 2017). (b) Τhe eigendecomposition of the adjacency (left plot) and normalised Laplacian (right plot) matrix revealed the related eigenvalues, and it is linked to the spectral analysis of a graph like the Fourier transform of a time series. The smallest (most positive) Laplacian eigenvalues (labelled in lilac) are associated with low‐frequency modes of the graph (c, top brain view). In contrast, the largest (most negative) Laplacian eigenvalues (or most negative adjacency eigenvalues) (labelled in pink) are associated with high‐frequency modes (c, bottom brain views). Both modes define the graph Fourier transform (GFT). Functional MRI data or EEG or MEG measured at every single ROI representing the nodes of the graph (network) (d) can be decomposed employing these modes and can be transformed through graph signal processing (GSP) tools (e). Topographies have been created with BrainNet Viewer (Xia et al., 2013)

Graph signal processing (GSP) is an emerging research area that combines a graph structure with brain signals $x\in R^{N}$ interpreting brain signal x as graph signals encapsulating the topology of a brain graph. Brain networks are modelled via a weighted brain graph $G=\left(V,A\right)$ where $V=\left\{1,2,\ldots ,N\right\}$ is a set of N nodes associated with specific brain regions and are directly related to the adopted atlas that parcellates brain volume into distinct brain regions and $A\in ⥂R^{N\times N}$ is a weighted adjacency matrix with entries $A_{\mathrm{ij}}$ representing the functional strength between brain regions i and j or characteristic estimated measures related to tracts in diffusion MRI (Figure 13).

GSP has been introduced to employ brain graph to analyse brain signals x. For weighted brain graph with only positive weights, we can either adopt the original adjacency matrix A or the graph Laplacian L=D−A where the D matrix is a matrix with dimensions N×N as the adjacency matrix A with all the entries equal to 0 except for the main diagonal that tabulates the degree of every node (Chung, 1997). The degree of every node refers to the total number of existing connections of every node, with the rest of the N−1 nodes ranging from 1 up to N−1. Alternative Laplacian operators are: the symmetric normalised graph Laplacian $L_{\mathrm{sym}}=D^{-1/2}LD^{-1/2}$ that smooths the differences in degree enhancing the contribution of the connectivity tabulating in A or a random‐walk normalised graph Laplacian $L_{\mathrm{rw}}=D^{-1}L$ (Von Luxburg, 2007).

We can define a graph shift operators S assuming that S is diagonalizable using singular value decomposition (SVD), such as $S={V\Lambda V}^{-1}$ where Λ is a diagonal matrix containing the eigenvalues, and V its eigenvectors. When V is symmetric, then V is real and unitary, implying that $V^{-1}=V^{T}$. The main goal of the definition of S as a graph shift operator is to build a transformation process that characterises exchanges of information between neighbouring nodes. The eigendecomposition of S is then used to define the graph spectral domain.

Consider a signal $x\in R^{N}$ and a graph shift operator $S={V\Lambda V}^{-1}\in R^{N\times N}$ decomposed on its eigenvalues and eigenvectors, then the following pair of transformations form the graph Fourier transform (GFT) and the inverse graph Fourier transform (iGFT):

(4) $$x^{\prime }=V^{T}x\;\text{and}\;x=\mathrm{Vx}\prime .$$

where $x^{\prime }=\left[x_{1}^{\prime },x_{2}^{\prime }\ldots ,x_{n}^{\prime }\right]$ can be thought of as the graph frequencies of the signal (Shuman et al., 2013).

The first Laplacian eigenvalues reflect the brain network's community structure, with the smaller first eigenvalues supporting a strong community structure. Explicitly repeated eigenvalues reflect motif duplications or additions, while the largest eigenvalue is associated with the bipartiness level of the most bipartite sub‐network (de Lange et al., 2014).

The smallest Laplacian eigenvalues (or most positive adjacency eigenvalues) are associated with low‐frequency modes on the graph. In contrast, the largest Laplacian eigenvalues (or most negative adjacency eigenvalues) are associated with high‐frequency modes. These modes define the GFT. The GFT encodes the graph signal variability, such as the Fourier transform encodes for temporal brain signals. Brain signals linked to specific brain graph nodes related to specific brain areas can be decomposed using those nodes and transformed by employing tools from graph signal processing.

Discrete Fourier transform (DFT) has also been introduced to tailor graphs as in signals by considering cycle graphs. Cycle graphs tabulated the number of cycles between every pair of nodes representing discrete periodic signals (Leonardi and Van De Ville, 2013). For this graph, the eigenvectors of its adjacency $A_{\text{cycle}}$ or its Laplacian matrix $L_{\text{cycle}}=2I-A_{\text{cycle}}$ satisfy V=F. Since cycle graphs represent discrete periodic signals, a GFT is equivalent to a DFT for cyclic graphs. We also note that it is possible to combine DFT and GFT to investigate the joint spatial–temporal frequency, that is, $X^{\prime \prime }=V^{T}XF^{H}$ where F refers to the Fourier matrix tabulates the Fourier coefficients, and t·H indicates the Hermitian transpose of the F (Huang et al., 2018).

## 8 THE RELATIONSHIP OF CONNECTOMICS AND CHRONNECTOMICS TO COGNITION AND BEHAVIOUR

In the majority of neuroimaging studies, numerous behavioural, cognitive and neuropsychological measures are estimated. These measures should be first pre‐filtered to reveal the ones that share complementary information and avoid the multicollinearity effect. At a second level, canonical correlation analysis could be adopted to find modes of optimal covariation between connectomics and behaviour‐demographics (Smith et al., 2015).

## 9 COMPARATIVE CONNECTOMICS ACROSS SPECIES

Connectomics and chronnectomics can be used in various ways by comparing their consistency across multiple sites, across neuroimaging systems, and across species (van den Heuvel et al., 2016). It is important for evolutionary neurobiologists and geneticists to compare the derived connectomic maps across various species except for monkeys, cats, rats and C. Elegans. For that purpose, comparative studies should adopt a similar acquisition methodology to map and explore connectomics' differences across species. This module aspires to become a common framework for many research studies that will aim to apply for such comparisons.

## 10 APPLICATION TO EXPERIMENTAL FMRI DATA

As an application and immediate demonstration of the presented module, we considered the data set MyConnectome; a data set comprised of an array of psychological and biological recordings of a single healthy human over 532 days. We focused on the 84, 10 min long, resting‐state fMRI scans. Each volume contains 640 voxels that make up the following 12 networks: Cingulo Opercular, Default Mode Network, Dorsal Attention, Frontoparietal 1, Frontoparietal 2, Medial Parietal, Parieto Occipital, Salience, Somatomotor, Ventral Attention, Visual 1, Visual 2. An overview of the study design, analysis pipelines and data acquisition parameters can be found elsewhere (Poldrack et al., 2015). We used our module in a well‐defined pipeline alongside other important scientific modules in a complimentary manner.

Our pipeline's backbone is based on the computation of dynamic functional connectivity using a sliding window over the fMRI time series and estimating the connectivity. For demonstration reasons, we opted to use the classic Pearson's correlation. The sliding window's width was set to 20 samples, and each iteration is moved forward 1 sample. The connectivity is estimated within this sliding window, resulting in 101 connectivity matrices (temporal segments×ROIs×ROIs=101×640×640) per scan.

Furthermore, for each connectivity profile, at first, we estimated the nodal global efficiency (NGE). Then, a mean global efficiency (GE) has been estimated from the ROIs belonging to the same brain sub‐network. This approach leads to a 2D matrix of size 12×8484, where 12 are the brain networks, and 8484=scans*temporal segments=84*101. This integrated 2D matrix that encapsulates all the important information across scans and temporal segments were projected into a common two‐dimensional space using Uniform Manifold Approximation and Projection for Dimension Reduction (UMAP). Then, we clustered the projected manifold into a small number of prototypes using Neural Gas (NGAS).

**FIGURE 14:**
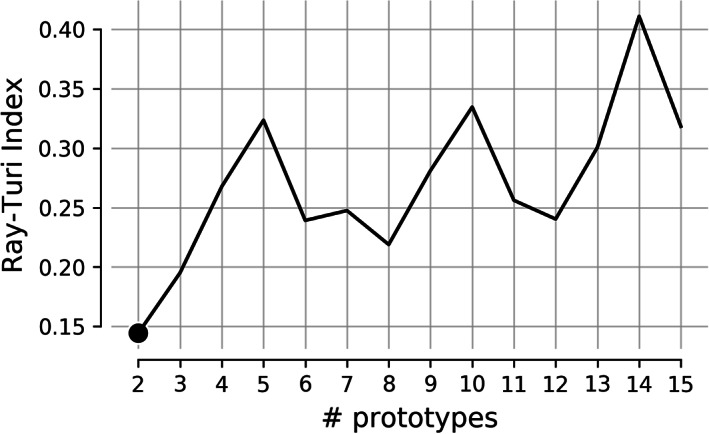
The Ray Turi index for measuring a clustering's compactness

**FIGURE 15:**
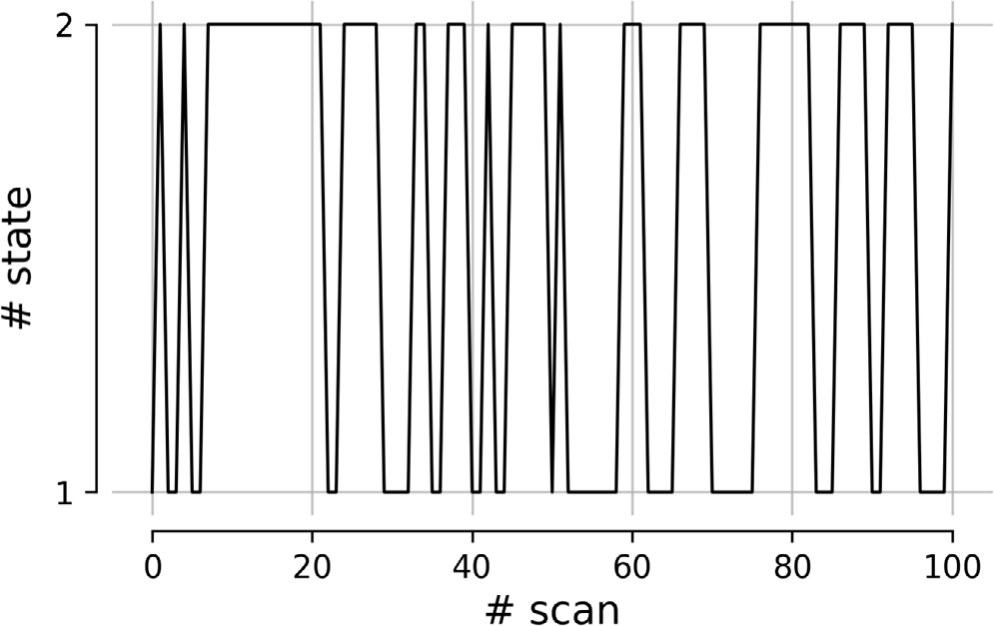
Temporal Evolution of Brain States across experimental time from the first scan

**FIGURE 16:**
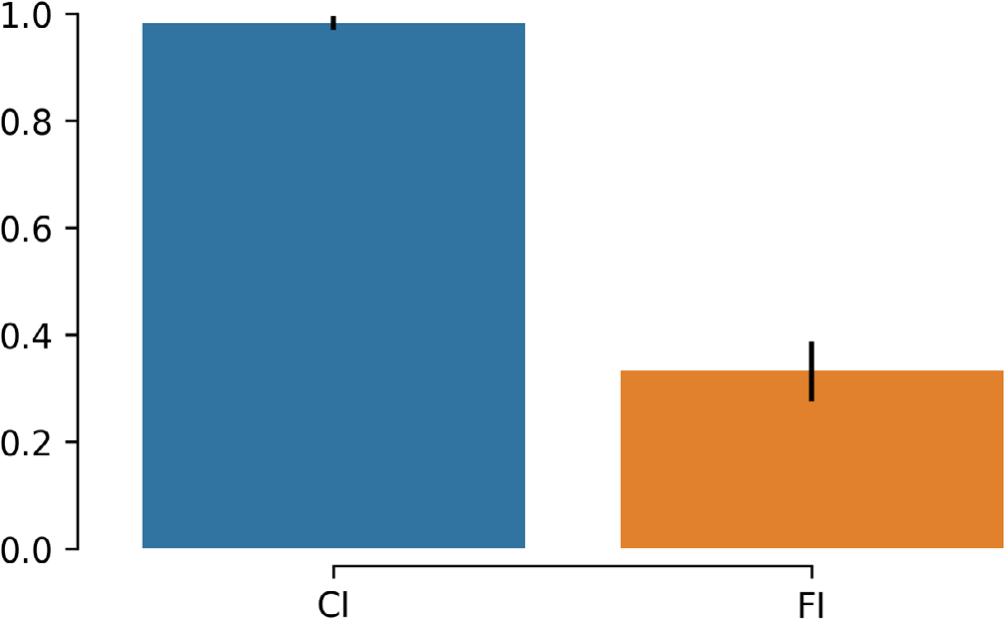
The mean and *SD* of Complexity Index and Flexibility Index across repeat scans

**FIGURE 17:**
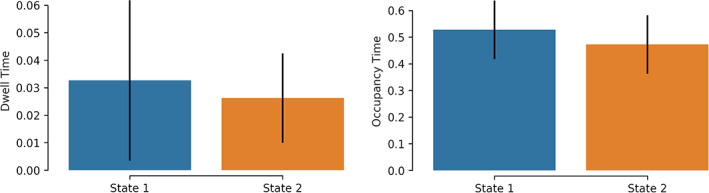
The mean and *SD* of Dwell Time and Occupancy for each state across scans

**FIGURE 18:**
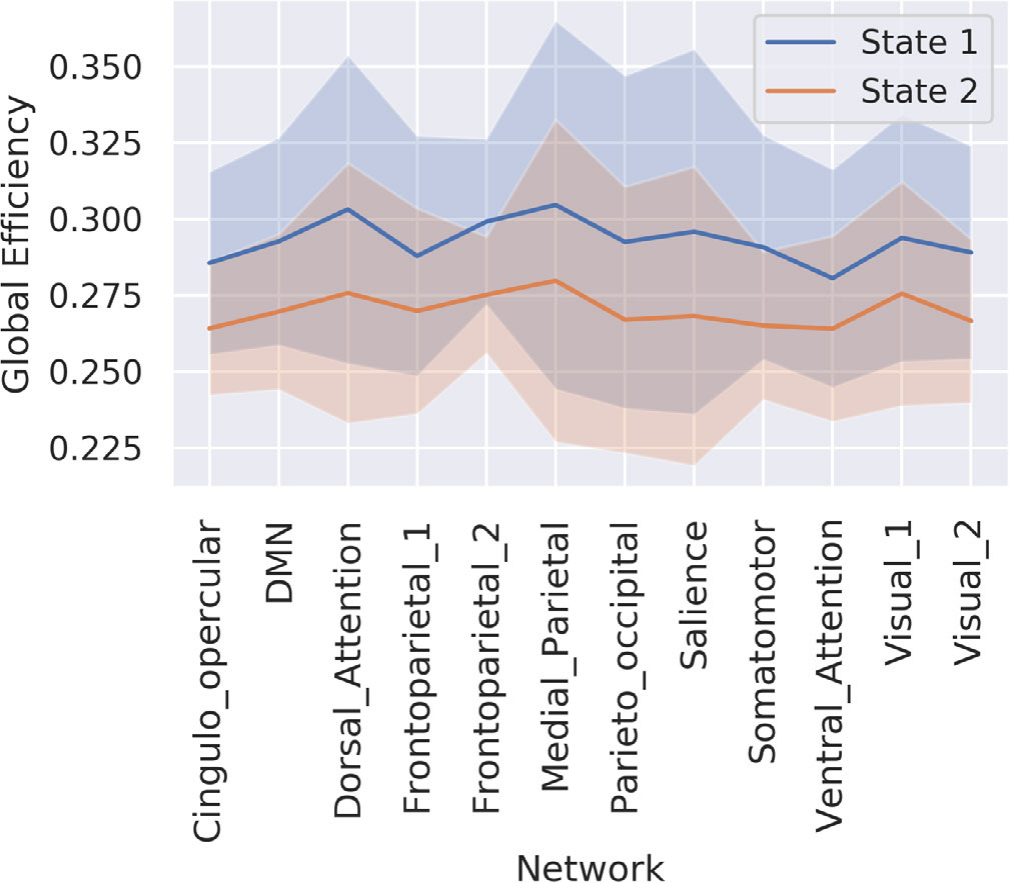
Mean and *SD* of global efficiency estimated over ROIs within each of the 12 brain subnetworks. *SD* is estimated across scans corresponding to each brain state

We concluded to use k=2 prototypes based on the Ray‐Turi criterion (Figure 14). From the symbolic time series presented in Figure 14 from the first scan, we computed the chronnectomics features associated with them (Figure 15). More specifically, we computed the Flexibility Index, Complexity Index, Dwell Time and Occupancy Time. Mean and *SD*s have been estimated across scans (Figures 16 and 17). Figure 18 illustrated the mean and *SD* of GE on the network level for both brain states. Our results align with the findings of a recent study on the same data set confirming further the existence of two brain states (metastates) that intermittently fluctuate over experimental and longitudinal time. They reported that these two distinct temporal brain states are associated with attentional resources (Shine et al., 2016).

## 11 COMPARING AND CLASSIFYING BRAIN NETWORKS

Complex network analysis is helpful to compare brain networks between conditions, between groups and across lifetime in longitudinal studies. There are two mainstream approaches to compare brain networks. The first approach includes a graph matching approach using graph distance metrics, graph edit distance metrics. Alternatively, another approach is based on the statistical comparison at the global level, edgewise, nodewise and spectral graph analysis via the Laplacian matrix's eigenanalysis. Our module provides the basic approaches of the aforementioned tools with a broad set of histogram distance metrics to compare nodal network metrics distribution, edge weights distribution and normalised Laplacian eigenvalues across any potential comparison scenario. Similarly, we adjusted both the k‐NN classifier and support vector machines properly to deal with either histograms or distributions and brain networks (2D matrices) (Dimitriadis et al., 2015). Histogram distance metrics like chi‐square and graph‐diffusion distance metrics are some of the kernels that can improve both classifiers' performance.

## 12 BRAIN CONNECTIVITY ANALYSIS MODULE

The landscape of neuroimaging tools, toolboxes and modules is vast and can be complicated. As brain connectivity became a widely accepted approach, virtually all available options provide connectivity estimators and measures. Multiple connectivity based and machine learning analysis software are freely available from various labs. A non‐exhaustive list of the most prominent neuroimaging python module includes nilearn (most methods are described in Abraham et al., 2014), PyMVPA (Hanke et al., 2009), scikit‐learn (Pedregosa et al., 2011) and MNE (Gramfort et al., 2013). Scikit‐learn is the de‐facto module for machine learning in python. Nilearn, provides a comprehensive framework for analysing brain volumes; it leverages scikit‐learn to provide statistical and machine‐learning methods. PyMVPA (MultiVariate Pattern Analysis in Python) is a package intended for statistical learning on large data sets. MNE, is a MEG and EEG specific analysis and visualisation toolbox; it includes both spectral and effective connectivity measures. Additionally, PACTOOLS (La Tour et al., 2017) is a set of tools to estimate the phase‐amplitude coupling in neural time series. Another noteworthy data analysis project is neuropycon (Meunier et al., 2019). It provides a framework to construct python‐based pipelines with a focus on connectivity and graph analysis. There are numerous toolboxes for MATLAB; some of which include PRoNTo (Schrouff et al., 2013), GraphVar (Kruschwitz et al., 2015, Waller et al., 2018), CONN (Whitfield‐Gabrieli & Nieto‐Castanon 2012), Fieldtrip (Oostenveld et al., 2011) and HERMES (Niso et al., 2013). PRoNTo and GraphVar are GUI toolboxes that can perform pattern recognition analysis of neuroimaging data. The functional connectivity toolbox (Zhou et al., 2009) is dedicated to fMRI modality, including high‐order network construction and classification. CONN implements the component‐based noise correction method (CompCor); a physiological and noise reduction strategy that filters first and second‐level connectivity analyses. It is based on SPM to provide a complete pipeline. Fieldtrip supports several connectivity measures with the focus being MEG, EEG and iEEG. HERMES is designed to study functional and effective brain connectivity from neurophysiological data such as multivariate EEG and/or MEG. It also includes visualisation tools and statistical methods to address the problem of multiple comparisons. EEGLAB (Delorme & Makeig, 2004), the most famous MATLAB toolbox for EEG data analysis, also provides a wide array of connectivity estimators with a convenient GUI. Regarding our proposed module, several features distinguish it from other software packages. First and foremost, our module is focused specifically on dynamic connectivity, chronnectomics and graph theory (thresholding, networks, multilayer networks, and graph distances). In addition, our module is developed with ‘mix and matching’ in mind; one can import only the functions they need. Regarding modalities, our module can process any type of modality data (EEG; MEG; fMRI; ECoG; fNIRS), as long as they can be represented in a standard NumPy array format. Subsequently, one can construct any available type of brain network (low‐order, high‐order, associated high‐order, multi‐layer network, integrated network, edge‐to‐edge) adapting a high number of functional connectivity estimators. Moreover, we provided the first set of a complete set of implemented CFC estimators. Our recent dominant coupling model is also available to build the integrated dynamic functional connectivity graph (iDFCG) that keeps both the strength and the type of interaction between every pair of brain areas and across experimental time. This set is not available yet, in other software. This iDFCG is accompanied by novel features tailored to this type of network. Moreover, we provided a complete set of histogram and graph matching metrics for comparing brain networks of the same modality or between structural and functional brain networks. This is another feature of our module that makes it unique among the others. These histogram and graph distance metrics have been introduced in two important classifiers, the k‐NN and support vector machines, to use these features on their original feature space and format instead of vectorizing them. This feature is also first represented in our module. Surrogates tailored to identify significant interactions and features tailored to multi‐layer networks have also been introduced. Statistical filtering of brain networks is also accompanied by a complete set of topological filtering algorithms that include both arbitrary and data‐driven. Our module's core is a complete set of mainstream pipelines for the definition of brain states from a DFCG. The estimation of famous chronnectomics accompanies the mining of DFCG, for example, occupancy time, dwell time, transition rate. Our module is open‐source software that allows researchers to customise their pipeline according to their needs, hypotheses and goals and incorporate any function into large‐scale analytic protocols. As with all available toolboxes and python modules, critical thinking and scientific reasoning is required when designing an experiment and selecting the methods.

## 13 CONCLUSION

In this article, we presented a python module, called dyconnmap, for static and dynamic brain network construction in multiple ways, mining time‐resolved function brain networks, network comparison and brain network classification. This module introduces the integration of state‐of‐the‐art signal processing analysis under the notion of within and between frequency coupling (CFC), the construction of brain networks in alternative scenarios. It furthermore provides a framework for comparing and classifying brain networks and their extracted features. The target audience is users with an understanding of every concept of algorithmic analysis and clinicians with domain knowledge but with less familiarity with writing customised software.

Dynamic functional connectivity analysis has emerged as an effective approach to characterise how associations between brain areas change over time. Here, we described an extensive collection of different types of connectivity estimators for both within‐ and between‐frequency coupling, the design of appropriate surrogates to detect significant interactions, and topological filtering algorithms to filter out a dense brain network. Mining time‐resolved functional brain networks are the central core of this module with the estimation of the essential chronnectomics features such as occupancy time, dwell time and transition rate that characterised the (dis)appearance of brain states evolved over experimental time. To the best of our knowledge, our module is the most comprehensive module where one can analyse time series extracted from specialised toolboxes tailored to every modality till the outcome and the demonstration of the key findings.

## Data Availability

Data sharing is not applicable to this article as no new data were created or analyzed in this study.

## APPENDIX A

### A.1 Module structure

Our proposed module, dyconnmap, is organised in multiple submodules. Each submodule groups together appropriate methods. The most fundamental submodules are fc, chronnectomics and graph. These submodules' relation and function are apparent; estimate the connectivity, compute the similarity between the connectivities, and extract chronnectomics features. There are numerous other submodules within the module, such as ‘ts’, for time series and symbolic time series analysis and ‘cluster’ for clustering methods and measuring the clusters' quality.

Tables A1, A2, A3, A4, A5

**TABLE A1:** Connectivity estimators and Surrogates analysis methods (found in the ‘fc’ and ‘ts’ submodules)

Method	Short description
Coherence (COH)	It is a measurement of two signals' linear relationship at a specific frequency (Nolte et al., 2004).
Correlation (CORR), partial and cross‐correlation (PART/CROSS)	Pearson's correlation, cross and partial (Friston et al., 1994).
Cosine (COS)	The cosine representation of the BOLD signals was recently used (Vohryzek et al., 2020) to bridge together dynamic systems theory and spontaneous brain activity.
Directed phase lag index (dPLI)	Directed Phase Lag Index was introduced (Stam & van Straaten, 2012) to capture the phase and lag relationship as a measure of directed functional connectivity
Imaginary coherence (ICHOH)	The imaginary part of coherence is insensitive to volume conduction artefacts (Nolte et al., 2004).
Imaginary part of phase locking value (IPLV)	It was proposed as an alternative to PLV to resolve its sensitivity to volume conduction and common reference effects (Sadaghiani et al., 2012).
Mutual information (MI)	(Vinck et al., 2011)
Phase lag index (PLI)	Phase lag index was developed as an alternative phase synchronisation estimator that is less prone to the effects of common sources (namely, volume conduction and active reference electrodes) (Stam et al., 2007).
Phase locking value (PLV)	Possibly the most well know phase estimator (Lachaux et al., 1999).
Weighted phase lag index (wPLI) and debiased weighted phase lag index (dwPLI)	Another alternative method to PLI to combat its noise and noise conduction effects (Vinck et al., 2011).
Surrogates, AAFT	Surrogates and their multiple approaches have been extensively described and studied (Theiler et al., 1992; Schreiber and Schmitz, 2000).

**TABLE A2:** Cross frequency coupling methods (from the ‘fc’ submodule)

Method	Short description
Phase amplitude coupling (PAC)	The most famous and prominent approach for studying the cross‐frequency coupling between slow and faster oscillations. The phase of a low frequency drives the power of a higher frequency.
Power‐envelope correlation (PEC)	Similarly to AEC, we can use the following formula to estimate the correlations in power between the different frequency bands (Hipp et al., 2012).
Amplitude envelope correlation (AEC)	Estimates the coupling without phase coherence and even among different frequencies by computing the correlation coefficient of a signal's amplitude envelope (Bruns et al., 2000).
Envelope‐to‐signal correlation (ESC)	It is similar to Amplitude Envelope Correlation, but the lower frequency oscillation amplitude is signed; thus, the phase information is preserved (Bruns et al., 2004).
Amplitude‐normalised envelope‐to‐signal correlation (NESC)	Like ESC, but it introduces the cosine of the signal, and so it is invariant to amplitude modulations and hence to co‐modulations in amplitude (Penny et al., 2008).
General linear model	The general linear model is used widely to detect coupling between a low and higher frequency (Penny et al., 2008; Penny et al., 2011).

**TABLE A3:** Brain states extraction (‘cluster’ submodule)

Method	Short description
Neural gas (NG)	An unsupervised adaptive algorithm that does not assume a preconstructed lattice, thus the adaptation cannot be based on the distances between the neighbour neurons because, by definition, there are no neighbours (Martinetz & Schulten, 1991).
Growing neural gas (GNG)	A dynamic neural network that learns topologies; compared with Neural Gas, GNG provides the functionality of adding or purging the constructed graph of nodes and edges when specific criteria are met (Fritzke, 1995).
Relational neural gas (RNG)	Relational Neural Gas is a variant of Neural Gas that allows clustering and mining data from a pairwise similarity or dissimilarity matrix (Hammer & Hasenfuss, 2007).
Merge neural gas (MNG)	It is similar to the original neural gas algorithm, but each node has an additional context vector associated; and the best matching unit is determined by a linear combination of both the weight and context vector (thus the merge), from the previous iteration (Strickert & Hammer, 2003).
Self‐organised maps (SOM)	One of the most famous clustering algorithms. It uses a neighbouring function to preserve the topological properties of the given input data (Kohonen, 2001)
Ray‐Turi	A validity measure developed to automatically (and data‐driven) find the number of clusters. It is based on the intra‐cluster and inter‐cluster distance measures (Ray & Turi, 1999).
Davies‐Bouldin	An index to measure the similarity of (any number) clusters (Davies & Bouldin, 1979).

**TABLE A4:** Chronnectomics**‐**related methods

Method	Short description
Dwell‐time (DT)	Dwell time measures the time (when used in functional connectivity microstates) in which a state is active consecutive temporal segments. (Dimitriadis et al., 2019)
Flexibility index (FI)	In the context of graph clustering, flexibility is the frequency of a node change module allegiance, the transition of brain states between consecutive temporal segments. The higher the number of changes, the larger the FI will be. (Bassett et al., 2011)
Occupancy time (OT)	The fraction of number of distinct symbols occurring in the symbolic time series (Dimitriadis et al., 2019).
Markov matrix	Markov matrix (also referred to as ‘transition matrix’) is a square matrix that tabulates the observed transition probabilities between symbols for a finite Markov Chain. It is a first‐order descriptor by which the next symbol depends only on the current symbol (and not on the previous ones); a Markov Chain model (Dimitriadis et al., 2019).
Transition rate (TR)	The total sum of transition between symbols (Dimitriadis et al., 2019).

**TABLE A5:** Methods available in the ‘graph’ submodule

Method	Short description
Graph diffusion distance (GDD)	The GDD metric is a measure of distance between two (positive) weighted graphs based on the Laplacian exponential diffusion kernel. The notion backing this metric is that two graphs are similar if they emit comparable patterns of information transmission.
Ipsen‐Mikhailov distance (IMD)	Given two graphs, this method quantifies their difference by comparing their spectral densities. This spectral density is computed as the sum of Lorentz distributions (Ipsen, 2004; Donnat & Holmes 2018).
Laplacian energy (LE)	Laplacian graph energy is a measure of graph complexity (Gutman, 2006).
Normalised mutual information (NMI)	Normalised Mutual Information proposed (Strehl & Ghosh, 2002) as an extension to Mutual Information to enable interpretations and comparisons between two partitions.
Multilayer participation coefficient (MPC)	Multilayer Participation Coefficient method from Guillon (Guilon et al., 2017) using the γ coefficient.
Nodal global efficiency (NGE)	The global efficiency as computed per node (Latora & Marchiori, 2001; Latora & Marchiori, 2003)
Spectral Euclidean distance (SED)	The spectral distance between graphs is simply the Euclidean distance between the spectra (Wilson & Zhu, 2008).
Spectral K distance (SKD)	Given two, we can use their k‐largest positive eigenvalues of their Laplacian counterparts to compute their distance (Jakobson & Rivin, 2000; Rincombe, 2006).
Variation of information (VI)	Variation of Information (Meilă, 2007) is an information‐theoretic criterion for comparing two partitions. It is based on the classic notions of entropy and mutual information. In a nutshell, VI measures the amount of information lost or gained in changing between two clusters. VI is a true metric, is always non‐negative and symmetric.
Threshold (k‐core decomposition, MST—Mean degree, mean degree, shortest paths, global‐cost efficiency, OMST global‐cost efficiency)	Global‐Cost Efficiency: threshold a graph based on the Global Efficiency ‐ Cost formula (Bassett et al., 2009). OMST Global‐Cost Efficiency: threshold a graph by optimising the formula GE‐C via orthogonal MSTs (Dimitriadis et al., 2017).

### A.2 Code availability

The code available under the ‘New BSD’ Licence at GitHub https://github.com/makism/dyconnmap. It supports Python 3.6 or newer than Python 3.8. One can find the complete source code, logs, installation instructions, hard and soft dependencies (such as NetworkX, NumPy, SciPy, scikit‐learn). Of course, no commercial software is required to use dyconnmap. In addition, because of Python's cross‐platform support, one can use dyconnmap virtually on every platform: Linux, Mac OS and Windows. In addition, finally, due to the very liberal licencing option, practically one can do as they please; there are as few as possible regulations.

Through PyPi (the Python Package Index—https://pypi.python.org, the official third‐party software repository), we make the distribution of our module robust and accessible practically to everyone with a standard python installation. The distributed files (called ‘wheels’ in the Python ecosystem) encapsulate the module itself (alongside the examples, tests, the licence file, etc.) and metadata, such as a list with the dependencies and target version. The installation through pip is as easy as typing the command ‘pip install dyconnmap’. Alternatively, if one uses the Anaconda distribution, the Anaconda channel must be specified; ‘pip instal ‐i https://pypi.anaconda.org/makism/simple dyconnmap’.

### A.3 Learning material

We have supplied the GitHub repository with numerous tutorials in verbose Jupyter notebooks format that cover all the aspects of our module. The tutorials are also available online through Binder (https://mybinder.org) at our interactive environment at https://mybinder.org/v2/gh/makism/dyconnmap/master?filepath=tutorials. Furthermore, there is an example snippet code almost for each function and method listed in the module.

### A.4 On development

On the development aspect of our project and following the spirit of other open‐source projects, we have hosted our module on GitHub. There, one can easily browse the source code and keep track of the changes made historically. For those interested in contributing to our project, GitHub provides a user‐friendly environment to make forks and create pull requests, which after review; they will be merged with the main public branch.

Moreover, we make use of third‐party applications integrated with GitHub, like Coveralls (https://coveralls.io) and Codacy (https://www.codacy.com). Coveralls are a web service that determines the code coverage of a project, to which degree the source code is executed given some tests. Coveralls report a code coverage of 93% for our project. Codacy, on the other hand, performs static analysis on the source code, reporting the quality of the code (i.e., unused variables or imported modules), the complexity of a piece of code, etc. Our project has been awarded a grade ‘A’ from Codacy for its quality.

To maintain the development progress in a forwarding momentum, we have integrated an online continuous integration solution, namely, TravisCI (https://travis-ci.org) to automate the code testing. The sole purpose of these services is to execute the provided test suites, given some criteria, that is, when a new piece of code is pushed to the repository or periodically. This way, we can keep track if a revised implementation of a method or algorithm produces the desired results. Furthermore, we are in the process of adjusting and updating the module to use the recently introduced ‘typing’ module. These ‘type hints’ (as they are called, see PEP 484) can be used with third‐party tools, that is, ‘mypy’ for type checking, or from IDE to provide useful feedback about the expected types of variables, functions parameters and return values. Our module evolves together with Python; Python 3.7 introduced the notion of ‘data classes’; a decorator that fills in standard methods in a class that is heavily used as a data container. We are working on a new ‘Data set’ class that utilises the data class feature to bind all the functionality provided in our module together, with built‐in parallel processing support (through a third‐party library, such as joblib or Numba). With the upcoming additions and improvements, we strive for transparency, reproducibility, and replicability.

Finally, considering the project's documentation, we have opted in for using ReadTheDocs (https://readthedocs.org/). An online platform to generate and produce HTML and PDF files from the source code's comments (of course, the same results can be achieved locally, just following the given instructions). This ensures that a manual textbook is generated automatically whenever the project is revised.

Please note that the specific links for the services described are maintained on the project's GitHub page.
